# Discovery of new chromen-4-one derivatives as telomerase inhibitors through regulating expression of dyskerin

**DOI:** 10.1080/14756366.2018.1466881

**Published:** 2018-08-22

**Authors:** Jie Quan Wang, Meng Di Yang, Xing Chen, Yang Wang, Liu Zeng Chen, Xiu Cheng, Xin Hua Liu

**Affiliations:** a School of Pharmacy, Anhui Province Key Laboratory of Major Autoimmune Diseases, Anhui Institute of Innovative Drugs, Anhui Medical University, Hefei, P. R. China;; b School of Material Science Chemical Engineering, ChuZhou University, ChuZhou, P. R. China

**Keywords:** Synthesis, chromen, anticancer activity, dyskerin, telomerase activity

## Abstract

A series of new trimethoxyphenyl-4H-chromen derivatives as telomerase inhibitors through regulation dyskerin were designed and synthesised. The anticancer activity assay *in vitro* showed that compound **5i** 3-(4-(4-isonicotinoylpiperazin-1-yl)butoxy)-5,7-dimethoxy-2-(3,4,5-trimethoxyphenyl)-4H-chromen-4-one exhibited high activity against Hela, SMMC-7721, SGC-7901, U87 and HepG2 cell lines. Compound **5i** also showed potent inhibitory activity against telomerase. The further results confirmed this title compound could significantly improve pathological changes induced rat hepatic tumor *in vivo*. Preliminary mechanisms showed that compound **5i** inhibited telomerase activity through decrease expression of dyskerin.

## 1. Introduction

Telomerase was found in most of human cancer specimens^1^. Among these binds, dyskerin plays a vital role^2^. As a nucleolus RNA-binding protein, dyskerin binds the TERC domain in telomerase holoenzyme and directs pseudouridylation of snRNAs. A lot of studies confirm that losing function of dyskerin influenced activity of telomerase in one hand. But, in the other hand, when lack of dyskerin, mRNA translation and factors of antiapototic synthesis were altered, such as bcl-2 and bcl-xl^3^ which lead cell cycle arrest or cell death^4^. Furthmore, dyskerin was also found to be associated with ribosome biogenesis^5^ and complex stabilisation of telomerase. Over-expression dyskerin linked to a variety of tumors^6–8^ have been found. Since dyskerin protein plays an essential role both in cellular and in individual processes; so, based on this process, novel highly selective compounds as telomerase inhibitors should be discovered for us^9–10^.

**Figure 1.**
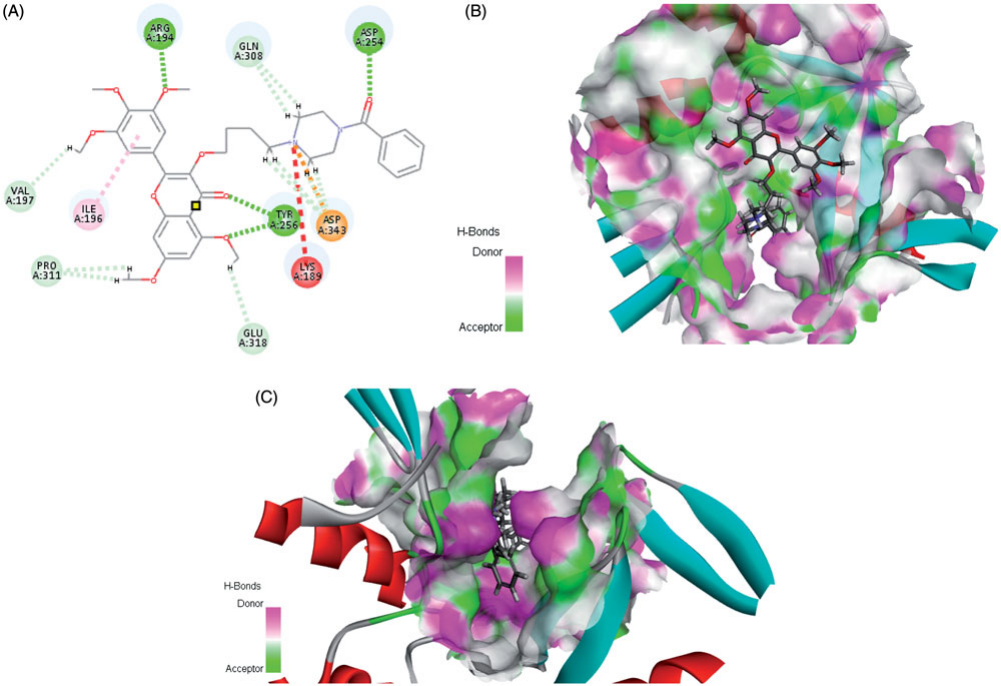
Rational design of target compound. (A) 2D picture of binding was depicted. (B) In order to reveal that the molecule was well filled in the active pocket, the enzyme surface was shown (Show all small molecule). (C) In order to reveal that the molecule was well filled in the active pocket, the enzyme surface was shown (Highlight phenyl-piperazine).

Ttrimethoxyphenyl-4H-chromen, a flavonoid compound, shows extensive anticancer activity^11–13^, which can decrease pancreatic cancer growth via induction of cell apoptosis^14–15^. It also can directly modulate the activity of a large number of important signaling molecules such as protein kinase^14^ and phosphatidylinositol 3-kinase^16–22^. Furthermore, this 4H-chromen compound could arrest human telomeric G-quadruplex structure as anticancer agent^23–26^. In our previous work, some analogs were designed as telomerase inhibitors, the preliminary structure–activity relationships (SARs) showed that introduction of six-membered ring containing nitrogen should be improve anticancer activity^27^. Since structural information of the binding site was available to us, the previous docking results also revealed that two amino acids LYS 189 and Asp 254 played vital roles in the conformation of bind^28^. So, we initiated an effort to provide guidance for this target active site. Is that what we expected, when piperazin-phenyl methanone moiety was introduced, the target compound could interact well with residues LYS 189 and Asp254 (Figure 1(A)). Furthermore, the cavity of the benzene ring has an optimised space (Figure 1(B and C)). Herein, in continuation to extend our research on chromen-4-one derivatives with anticancer activity, a series of new3–(4-(substitutebenzoylpiperazin-1-yl)butoxy)-5,7-dimethoxy-2–(3,4,5-trimethoxyphenyl)-4H-chromen-4-ones were designed and synthesised. Some compounds have good activities *in vivo* and *in vitro*. Preliminary Western blot results showed that the expressions of Bcl-2/Bax and PARP related to the function of dyskerin were reduced after treatment with title compound.

## 2. Experimental section

### 2.1. Chemistry

Used pre-coated silica GF254 plates, the reactions were monitored by TLC (thin-layer chromatography). Melting points were determined on a XT4MP apparatus (Taike Corp., Beijing, China). IR (KBr, cm^−1^) was recorded on NicoletFT-IR Spectrometer. ^1^H and ^13^C NMR spectra were recorded on a Brucker AM-500 (500 MHz) spectrometer, CDCl_3_ used as the solvent. Elemental analyses were performed on a CHN–O-rapid instrument and were within ±0.4% of the theoretical values.

With 5,7-dihydroxy-3-(3,4,5-trihydroxy-6-methyl-tetrahydro-2H-pyran-2-yloxy)-2-(3,4,5-trihydroxyphenyl)-4H-chromen-4-one as the raw material, 3-hydroxy-5,7-dimethoxy-2-(3,4,5-trimethoxyphenyl)-4H-chromen-4-one **2** was prepared through removing glycosides, detail procedure was follow: 2-(3,4-dihydroxyphenyl)-5,7-dihydroxy-3-(3,4,5-trihydroxy-6-methyl-tetra-hydro-2H-pyran-2-yloxy)-4H-chromen-4-one was dissolved in DMF, K_2_CO_3_ used as absorb acid agent, stirred for 10 min, methyl iodide was added, at room temperature for 48 h, washed with ethyl acetate, combined organic phase, then dissolved in ethanol, refluxed, added 5% hydrochloric acid, slowly, yellow solids were precipitated. Compound **2** (3 mmol) was dissolved in DMF (30 ml), K_2_CO_3_ (12 mmol) used as absorb acid agent, 1,4-dibrombutane was added and reaction for 12 h. The mixture was dispersed with water and extracted with ethyl acetate three times, the crude residue was purified by chromatography on SiO_2_ (petroleum ether: ethyl acetate = 2:1, v/v) to give compound **3**. ^1^H NMR (CDCl_3_, 500 MHz) δ: 1.82-1.85 (*m*, 2H, CH_2_), 2.00–2.03 (*m*, 2H, CH_2_), 3.45 (*t*, *J* = 11.5 Hz, 2H, CH_2_), 3.87–3.91 (*m*, 15H, 5 × OCH_3_), 3.99 (*t*, *J* = 10.3 Hz, 2H, CH_2_), 6.30 (d, *J* = 2.3 Hz, 1H, H-6), 6.45(d, *J* = 2.3 Hz, 1H, H-8), 7.30 (overlapping *s*, 2H, H-2′, H-6′); ^13^CNMR (CDCl_3_, 125 MHz) δ: 174.0 (C-4), 164.0 (C-7), 161.0 (C-9), 158.8 (C-2), 153.0 (C-5), 152.7 (C-3′, C-5′), 140.5 (C-4′), 139.9 (C-3), 126.0 (C-1′), 109.4 (C-10), 105.8 (C-6′, C-2′), 95.9 (C-6), 92.4 (C-8), 71.1 (OCH_2_), 61.1(4′-OCH_3_), 56.5 (3′, 5′, 7-OCH_3_), 55.9 (8-OCH_3_), 34.1 (CH_2_Br), 29.4 (CH_2_), 28.7 (CH_2_).

To a solution of DMF (50 ml), compound **3** (2.0 mmol) and K_2_CO_3_ (10 mmol) were added under room temperature, and then anhydrous piperazine (10 mmol) was added, reaction at room temperature for 12 h, the reaction was stopped, washed with water 30 ml × 3 and extracted with ethyl acetate three times (30 ml × 3). The crude residue was purified by chromatography on SiO_2_ (chloroform: methanol = 15:1, v/v) to give compound **4** as colourless solids, yield, 64.2%, m.p. 107 ∼ 109 °C. Compound **4:** IR (KBr, cm^−1^): *ν*_max_ 1627, 1602, 1558, 1506, 1417, 1350, 1244, 1211, 1128, 1014, 852, 815; ^1^H NMR (500 MHz, CDCl_3_) δ: 7.35 (2H, overlapping *s*, 2′-H, 6′-H), 6.48 (1H, d, *J* = 2.3 Hz, 8-H), 6.34 (1H, d, *J* = 2.3 Hz, 6-H), 4.03 (2H, *t*, *J* = 13.15 Hz, OCH_2_), 3.93 (15H, *m*, 5OCH_3_), 2.87 (4H, *t*, *J* = 9.75 Hz, piperazine), 2.36 (1H, br, piperazine), 2.31 (2H, *t*, *J* =15.5 Hz, CH_2_), 1.87 (4H, brs, piperazine), 1.73 (2H, *m*, CH_2_), 1.57 (2H, *m*, CH_2_); ^13^C NMR (125 MHz, CDCl_3_) δ: 174.1 (C-4), 164.0 (C-7), 161.1 (C-9), 158.8 (C-2), 153.0 (C-3′, C-5′), 152.5 (C-5), 140.8 (C-4′), 139.8 (C-3), 126.2 (C-1′), 109.5 (C-10), 105.9 (C-2′, C-6′), 95.8 (C-6), 92.4 (C-8), 72.4 (OCH_2_), 61.1 (4′-OCH_3_), 58.9 (CH_2_), 56.5 (7-OCH_3_), 56.4 (3′, 5′-2OCH_3_), 55.9 (5-OCH_3_), 54.3 (2CH_2_), 45.9 (2CH_2_), 28.4 (CH_2_), 23.1 (CH_2_).

### 2.2. Synthesis of title compounds 5a∼5l

**Scheme 1.**
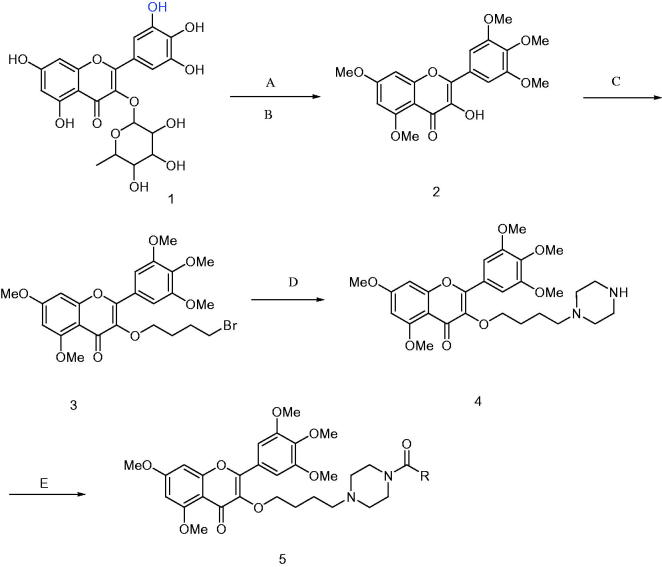
Synthesis of compounds **5a**∼**5l**. **5a**: R = 2,4-difluorobenzoyl **5b**: R = 2,6-difluorobenzoyl **5c**: R = 2-chloro-6-fluorobenzoyl **5d**: R = 3-fluorobenzoyl **5e**: R = 2-nitrobenzoyl **5f**: R = 4-trifluoromethyl **5g**: R = 4-nitrobenzoyl **5h**: R = 4-chlorobenzoyl **5i**: R = isonicotinoyl **5j**: R = benzoyl **5k**: R = 3,5-dinitrobenzoyl **5l**: R = 5-chloro-2-nitrobenzoyl. Reagent and conditions: (A) K_2_CO_3_, DMF, CH_3_I, r.t. 48 h; (B) 5% HCl, 95% ethanol, reflux, 2 h; (C) Br(CH_2_)_4_Br, acetone, K_2_CO_3_, reflux, 12 h; (D) K_2_CO_3_, DMF, Piperazine, r.t. 12 h; (E) HATU, DCM, **R**COOH, r.t. 12 h.

To a dry dichloromethane (20 ml) solution of carboxylic acid (2.0 mmol), HATU (2.4 mmol) and 99% triethylamine (2.0 ml) were added under the ice bath, stirred for 30 min. And then removed the ice bath, 5,7-dimethoxy-3–(4-(piperazin-1-yl)butoxy)-2–(3,4,5-trimethoxyphenyl)-4H-chrome-4-one (compound **4)** was added (2 mmol), reaction at room temperature for 12 h, TLC tracked (Chloroform: Methanol = 5: 1, v/v), the reaction was stopped, washed with water 30 ml × 3, dry with anhydrous sodium. The crude residue was purified by chromatography on SiO_2_ (chloroform: methanol = 10:1, v/v) to give compound **5** as light yellow solids (Scheme 1).

**5a:** Yield, 46.6%, m.p. 89 ∼ 91 °C; IR: *ν*_max_ 1625, 1600, 1558, 1506, 1417, 1350, 1244, 1211, 1128, 1016, 852, 821; ^1^H NMR δ: 7.38 (1H, *q*, *J* = 22.3 Hz, PhH), 7.33 (2H, overlap *s*, H-2’, H-6’), 6.93 (1H, *t*, *J* = 16.1 Hz, PhH), 6.83 (1H, *t*, *J* = 18.3 Hz, PhH), 6.48 (1H, d, *J* = 2.3 Hz, H-8), 6.34 (1H, d, *J* = 2.3 Hz, H-6), 4.01 (2H, *t*, *J* = 12.6 Hz, OCH_2_), 3.89–3.94 (15H, *m*, 5OCH_3_), 3.78 (2H, br, piperazine), 3.31 (2H, br, piperazine), 2.49 (2H, *m*, CH), 2.38 (4H, *m*, piperazine), 1.72–1.75 (2H, *m*, CH_2_), 1.63 (2H, br, CH_2_); ^13^C NMR δ: 174.1 (4-C), 164.3 (C=O), 164.1 (7-C), 161.1 (9-C), 158.9 (2-C), 153.0 (3′-C, 5′-C), 152.6 (5-C), 140.7 (4′-C), 140.0 (3-C), 130.7 (CH), 126.2 (1′-C), 112.2, 112.1, 109.5 (10-C), 106.0 (6′-C, 2′-C), 104.5, 104.3, 104.2, 95.9 (6-C), 92.5 (8-C), 72.2 (OCH_2_), 61.0 (4′-OCH_3_), 58.0 (CH_2_), 56.5 (7-OCH_3_), 56.4 (3′, 5′-2OCH_3_), 55.9 (5-OCH_3_), 53.2, 52.7, 47.0, 42.0, 28.2, 23.1. Anal. calcd for C_35_H_38_F_2_N_2_O_9_: C, 62.87; H, 5.73; N, 4.19%. Found: C, 63.15; H, 6.01; N, 4.45%.

**5b:** Yield, 52.1%, m.p. 96 ∼ 98 °C; IR: *ν*_max_ 1627, 1600, 1558, 1506, 1417, 1350, 1244, 1211, 1128, 1016, 852, 819; ^1^H NMR δ: 7.29–7.33 (3H, *m*, 2′-H, 6′-H, PhH), 6.92 (2H, *t*, *J* = 15.5 Hz, PhH), 6.47 (1H, d, *J* = 1.7 Hz, 8-H), 6.33 (1H, d, *J* = 2.3 Hz, 6-H), 4.01 (2H, *t*, *J* = 12.6 Hz, OCH_2_), 3.89–3.94 (15H, *m*, 5OCH_3_), 3.79 (2H, br, piperazine), 3.29 (2H, br, piperazine) , 2.46 (2H, *m*, CH_2_), 2.35 (4H, *m*, piperazine), 1.72 (2H, *m*, CH_2_), 1.60 (2H, *m*, CH_2_); ^13^C NMR δ: 174.1 (4-C), 164.0 (7-C), 161.1 (9-C), 160.0 (2C), 158.8 (2-C), 157.9 (C=O), 153.0 (3′-C, 5′-C), 152.6 (5-C), 140.7 (4′-C), 139.9 (3-C), 131.0, 130.9, 126.1 (1′-C), 111.9, 111.7, 109.4 (10-C), 106.0 (6**′**-C, 2′-C), 95.9 (6-C), 92.5 (8-C), 72.2 (OCH_2_), 61.0 (4′-OCH_3_), 58.0, 56.5 (7-OCH_3_), 56.4 (3′, 5′-2OCH_3_), 55.9 (5-OCH_3_), 53.2, 52.6, 46.8, 41.9 (CH_2_), 28.2 (CH_2_), 23.2 (CH_2_). Anal. calcd for C_35_H_38_F_2_N_2_O_9_: C, 62.87; H, 5.73; N, 4.19%. Found: C, 62.60; H, 6.10; N, 4.03%.

**5c:** Yield, 48.7%, m.p. 105 ∼ 107 °C; IR: *ν*_max_ 1625, 1600, 1558, 1506, 1417, 1350, 1246, 1211, 1130, 1014, 852, 815; ^1^H NMR δ: 7.32 (2H, overlap s, 2′-H, 6′-H), 7.24–7.29 (1H, *m*, PhH), 7.19 (1H, d, *J* = 8.1 Hz, PhH), 7.01 (1H, *t*, *J* = 16.6 Hz, PhH), 6.46 (1H, d, *J* = 2.3 Hz, 8-H), 6.31 (1H, d, *J* = 1.7 Hz, H-6), 3.99 (2H, *t*, *J* = 13.2 Hz, OCH_2_), 3.88–3.92 (15H, *m*, 5OCH_3_), 3.87 (2H, br, piperazine), 3.21 (2H, brm, piperazine), 2.45 (2H, *t*, *J* = 10.3 Hz, CH_2_), 2.31–2.34 (4H, *m*, H in piperazine), 1.69–1.71 (2H, *m*, CH_2_), 1.57 (2H, *m*, CH_2_); ^13^C NMR δ: 174.1 (4-C), 164.0 (7-C), 161.8 (C), 161.0 (9-C), 158.8 (2-C), 157.7 (C), 153.0 (3′-C, 5′-C), 152.6 (5-C), 140.7 (4′-C), 139.9 (3-C), 131.9 (CH), 130.8 (CH), 130.7 (CH), 126.2 (1′-C), 125.5 (CH), 114.5 (CH), 114.4 (CH), 109.5 (10-C), 106.0 (2′-C, 6′-C), 95.9 (6-C), 92.5 (8-C), 72.3 (OCH_2_), 61.0 (4′-OCH_3_), 58.0 (CH_2_), 56.5 (7-OCH_3_), 56.4 (3′, 5′-2OCH_3_), 55.9 (5-OCH_3_), 53.3 (CH_2_), 52.7 (CH_2_), 46.6 (CH_2_), 41.8 (CH_2_), 28.2 (CH_2_), 23.3 (CH_2_). Anal. calcd for C_35_H_38_ClFN_2_O_9_: C, 61.36; H, 5.59; N, 4.09%. Found: C, 61.64; H, 5.44; N, 4.42%.

**5d:** Yield, 51.2%, m.p. 117 ∼ 119 °C; IR: *ν*_max_ 1627, 1602, 1558, 1506, 1417, 1350, 1244, 1211, 1126, 1016, 852, 819; ^1^H NMR δ: 7.35 (1H, *m*, PhH), 7.32 (2H, overlap *s*, 2′-H, 6′-H), 7.14 (1H, d, *J* = 8.0 Hz, PhH), 7.07–7.09 (2H, *m*, Hz, PhH), 6.46 (1H, d, *J* = 2.3 Hz, 8-H), 6.31 (1H, d, *J* = 2.3 Hz, H-6), 4.00 (2H, *t*, *J* = 12.6 Hz, OCH_2_), 3.73–3.92 (15H, *m*, 5OCH_3_), 3.73 (2H, br, piperazine), 3.37 (2H, br, piperazine), 2.48 (2H, br, piperazine), 2.38 (2H, *m*, CH_2_), 2.34 (2H, br, piperazine), 1.70–1.73 (2H, *m*, CH_2_), 1.59–1.62 (2H, *m*, CH_2_); ^13^C NMR δ: 174.1 (4-C), 168.8 (C=O), 164.0 (7-C), 161.6 (C), 161.0 (9-C), 158.8 (2-C), 153.0 (3′-C, 5′-C), 152.6 (5-C), 140.7 (4′-C), 140.0 (3-C), 130.4 (CH), 130.3 (CH), 126.2 (1′-C), 122.8 (CH), 116.9 (CH), 114.5 (CH), 114.3 (CH), 109.5 (10-C), 106.0 (6′-C, 2′-C), 95.9 (6-C), 92.5 (8-C), 72.2 (OCH_2_), 58.0 (CH_2_), 56.5 (7-OCH_3_), 56.4 (3′, 5′-2OCH_3_), 55.9 (5-OCH_3_), 52.8 (CH_2_), 47.4 (CH_2_), 42.0 (CH_2_), 28.2 (CH_2_), 23.1 (CH_2_). Anal. calcd for C_35_H_39_FN_2_O_9_: C, 64.60; H, 6.04; N, 4.31%. Found: C, 64.29; H, 6.39; N, 4.00%.

**5e:** Yield, 54.3%, m.p. 141 ∼ 143 °C; IR: *ν*_max_ 1627, 1598, 1558, 1506, 1417, 1348, 1246, 1213, 1128, 1109, 1016, 852, 819; ^1^H NMR δ: 8.19 (2H, m, PhH), 7.58 (1H, d, *J* = 8.0 Hz, PhH), 7.33 (1H, overlap *s*, 2′-H, 6′-H), 7.25 (2H, *s*, Ph H), 6.46 (1H, d, *J* = 2.3 Hz, 8-H), 6.34 (1H, d, *J* = 1.7 Hz, 6-H), 4.01 (2H, *t*, *J* = 12.6 Hz, OCH_2_), 3.89–3.94 (15H, *m*, 5OCH_3_), 3.73 (2H, *m*, piperazine), 3.20 (2H, *m*, piperazine), 2.30–2.51 (6H, *m*, CH_2_ and piperazine), 1.71–1.73 (2H, *m*, CH_2_), 1.60 (2H, *m*, CH_2_); ^13^C NMR δ: 174.1 (4-C), 164.4 (C=O), 164.1 (7-C), 161.1 (C-9), 158.9 (2-C), 153.0 (3′-C, 5′-C), 152.6 (5-C), 146.7 (C), 140.7 (4′-C), 140.0 (3-C), 137.5 (CH), 137.3 (C),131.0 (CH), 126.2 (1’-C), 125.0 (CH), 123.3 (CH), 109.5 (10-C), 106.0 (6′-C, 2′-C), 95.9 (6-C), 92.5 (8-C), 72.2 (OCH_2_), 61.0 (4′-OCH_3_), 58.0 (CH_2_), 56.5 (7-OCH_3_), 56.4 (3′, 5′-2OCH_3_), 56.0 (5-OCH_3_), 53.1 (CH_2_), 52.6 (CH_2_), 46.8 (CH_2_), 42.0 (CH_2_), 28.2 (CH_2_), 23.2 (CH_2_). Anal. calcd for C_35_H_39_N_3_O_11_: C, 62.03; H, 5.80; N, 6.20%. Found: C, 61.82; H, 6.17; N, 5.89%.

**5f:** Yield, 65.7%, mp: 90 ∼ 92 °C; IR: *ν*_max_ 1625, 1602, 1558, 1506, 1417, 1350, 1243, 1211, 1128, 1016, 852, 817; ^1^H NMR δ: 7.65 (1H, d, *J* = 8.1 Hz, PhH), 7.56 (1H, *t*, *J* = 14.9 Hz, PhH), 7.48 (1H, *t*, *J* = 15.5 Hz, PhH), 7.32 (2H, overlap *s*, 2′-H, 6′-H), 7.25 (1H, d, *J* = 8.1 Hz, PhH), 6.46 (1H, d, *J* = 2.3 Hz, 8-H), 6.32 (1H, d, *J*= 2.3 Hz, 6-H), 4.00 (2H, *t*, *J* =12.6 Hz, OCH_2_), 3.88–3.93 (15H, *m*, 5OCH_3_), 3.75 (2H, br, piperazine), 3.11 (2H, *t*, *J* = 9.7 Hz, piperazine), 2.34–2.44 (2H, *m*, piperazine), 2.32 (2H, *t*, *J* = 14.9 Hz, CH_2_), 2.31 (2H, *m*, piperazine), 1.59 (2H, *m*, CH_2_), 1.57 (2H, *m*, CH_2_); ^13^C NMR δ: 174.1 (4-C), 167.3 (C=O), 164.0 (7-C), 161.0 (9-C), 158.8 (2-C), 153.0 (3′-C, 5′-C), 152.6 (5-C), 140.7 (4′-C), 139.9 (3-C), 134.9 (C), 132.2 (CH), 129.1(CH), 127.3 (CH), 126.7 (CH), 126.6 (C), 126.2 (1′-C), 109.5 (10-C), 106.0 (6′-C, 2′-C), 95.9 (6-C), 92.5 (8-C), 72.3 (OCH_2_), 61.0 (4′-OCH_3_), 58.0 (CH_2_), 56.5 (7-OCH_3_), 56.4 (3′, 5′-2OCH_3_), 55.9 (5-OCH_3_), 52.7 (CH_2_), 52.6 (CH_2_), 47.2 (CH_2_), 41.7 (CH_2_), 28.2 (CH_2_), 23.3 (CH_2_). Anal. calcd for C_36_H_39_F_3_N_2_O_9_: C, 61.71; H, 5.61; N, 4.00%. Found: C, 62.07; H, 6.00; N, 3.59%.

**5g:** Yield, 58.7%, m.p. 149 ∼ 151 °C; IR: *ν*_max_ 1627, 1598, 1558, 1506, 1417, 1348, 1246, 1213, 1128, 1109, 1016, 852, 819; ^1^H NMR δ: 8.27 (2H, d, *J* = 9.2 Hz, CH), 7.53 (2H, d, *J* = 9.2 Hz, CH), 7.32 (2H, overlap *s*, 2′-H, 6′-H), 6.47 (1H, d, *J* = 2.3 Hz, H-8), 6.32 (1H, d, *J* = 2.3 Hz, 6-H), 4.00 (2H, *t*, *J* = 12.6 Hz, OCH_2_), 3.88–3.93 (15H, *m*, 5OCH_3_), 3.74 (2H, br, piperazine), 3.30 (2H, br, piperazine), 2.46 (2H, br, piperazine), 2.34 (2H, *t*, *J* =14.9 Hz, CH_2_), 2.29 (2H, brm, piperazine), 1.69–1.72 (2H, *m*, CH_2_), 1.58–1.64 (2H, *m*, CH_2_); ^13^C NMR δ: 174.1 (4-C), 167.9 (C=O), 164.0 (7-C), 161.0 (9-C), 158.9 (2-C), 153.0 (3′-C, 5′-C), 152.6 (5-C), 148.4 (C), 142.1 (C), 140.7 (4′-C), 140.0 (3-C), 128.2 (2CH), 126.2 (1′-C), 123.9 (2CH), 109.5 (10-C), 105.9 (6′-C, 2′-C), 95.9 (6-C), 92.5 (8-C), 72.2 (OCH_2_), 61.0 (4′-OCH_3_), 58.0 (CH_2_), 56.5 (7-OCH_3_), 56.4 (3′, 5′-2OCH_3_), 55.9 (5-OCH_3_), 53.2 (CH_2_), 52.7 (CH_2_), 47.7 (CH_2_), 42.3 (CH_2_), 28.2 (CH_2_), 23.2 (CH_2_). Anal. calcd for C_35_H_39_N_3_O_11_: C, 62.03; H, 5.80; N, 6.20%. Found: C, 62.44; H, 5.77; N, 6.31%.

**5h:** Yield, 64.3%, m.p. 102 ∼ 104 °C; IR: *ν*_max_ 1633, 1600, 1558, 1506, 1417, 1348, 1244, 1213, 1126, 1109, 1008, 862, 819; ^1^H NMR δ: 7.33–7.37 (2H, *m*, 2’-H, 6’-H, PhH), 6.47 (1H, d, *J* = 2.3 Hz, 8-H), 6.33 (1H, d, *J* = 2.3 Hz, 6-H), 4.02 (2H, *t*, *J* = 12.6 Hz, OCH_2_), 3.88–3.94 (15H, *m*, 5OCH_3_), 3.72 (2H, br, piperazine), 3.36 (2H, br, piperazine), 2.45 (2H, br, CH_2_), 2.34 (2H, *t*, *J* = 14.4 Hz, CH_2_), 2.30 (2H, brm, CH_2_), 1.70–1.75 (2H, *m*, CH_2_), 1.58–1.60 (2H, *m*, CH_2_); ^13^C NMR δ: 174.1 (4-C), 169.2 (C=O), 164.0 (7-C), 161.1 (9-C), 158.9 (2-C), 153.0 (3′-C, 5′-C), 152.6 (5-C), 140.8 (4′-C), 140.0 (3-C), 135.8 (C), 134.2 (C), 128.8 (2CH), 128.7 (2CH), 126.2 (1’-C), 109.5 (10-C), 106.0 (6′-C, 2′-C), 95.9 (6-C), 92.5 (8-C), 72.2 (OCH_2_), 61.0 (4′-OCH_3_), 58.0 (CH_2_), 56.5 (7-OCH_3_), 56.4 (3′, 5′-2OCH_3_), 55.9 (5-OCH_3_), 53.4 (CH_2_), 52.9 (CH_2_), 47.7 (CH_2_), 42.3 (CH_2_), 28.3 (CH_2_), 23.2 (CH_2_). Anal. calcd for C_35_H_39_ClN_2_O_9_: C, 63.01; H, 5.89; N, 4.20%. Found: C, 62.88; H, 5.6.16; N, 4.29%.

**5i:** Yield, 65.5%, m.p. 81 ∼ 83 °C; IR: *ν*_max_ 1653, 1653, 1558, 1506, 1417, 1350, 1246, 1213, 1128, 1016, 819; ^1^H NMR δ: 8.57 (2H, d, *J* = 5.7 Hz, CH), 7.24 (2H, overlap *s*, 2′-H, 6′-H), 7.18 (2H, d, *J* = 5.7 Hz, CH), 6.39 (1H, d, *J* = 2.3 Hz, 8-H), 6.23 (1H, d, *J* = 2.3 Hz, 6-H), 3.92 (2H, *t*, *J* = 12.6 Hz, OCH_2_), 3.79–3.83 (15H, *m*, 5OCH_3_), 3.65 (2H, br, piperazine), 3.23 (2H, br, piperazine), 2.38 (2H, br, piperazine), 2.26 (2H, *t*, *J* = 14.9 Hz, CH_2_), 2.22 2H, (brm, piperazine), 1.62–1.66 (2H, m, CH_2_),1.49–1.54 (2H, *m*, CH_2_); ^13^C NMR δ: 174.0 (4-C), 167.5 (C=O), 164.0 (7-C), 160.9 (9-C), 158.7 (2-C), 152.9 (3′-C, 5′-C), 152.5 (5-C), 150.2 (2CH), 143.5 (CH), 140.6 (4′-C), 139.8 (3-C), 126.0 (1′-C), 121.3 (2CH), 109.3 (10-C), 105.8 (6′-C, 2′-C), 95.8 (6-C), 92.5 (8-C), 72.1 (OCH_2_), 60.9 (4′-OCH_3_), 57.9 (CH_2_), 56.4 (7-OCH_3_), 56.3 (3′, 5′-2OCH_3_), 55.9 (5-OCH_3_), 53.1 (CH_2_), 52.6 (CH_2_), 47.4 (CH_2_), 41.9 (CH_2_), 28.1 (CH_2_), 23.1 (CH_2_). Anal. calcd for C_34_H_39_N_3_O_9_: C, 64.44; H, 6.20; N, 6.63%. Found: C, 64.10; H, 5.81; N, 7.02%.

**5j:** Yield, 68.4%, m.p. 76  ∼ 78 °C; IR: *ν*_max_ 1627, 1602, 1558, 1506, 1417, 1348, 1242, 1211, 1128, 1008, 852, 819; ^1^H NMR δ: 7.37 (5H, *s*, PhH), 7.32 (2H, overlap *s*, 2′-H, 6′-H), 6.47 (1H, d, *J* = 2.3 Hz, 8-H), 6.33 (1H, d, *J* = 1.7 Hz, 6-H), 3.99 (2H, *t*, *J* = 12.1 Hz, OCH_2_), 3.88–3.93 (15H, *m*, 5OCH_3_), 3.80 (2H, br, piperazine), 3.45 (2H, br, piperazine), 2.47–2.55 (6H, *m*, CH_2_), 1.67–1.73 (4H, *m*, CH_2_); ^13^C NMR δ: 174.1 (4-C), 170.3 (C=O), 164.1 (7-C), 161.1 (9-C), 158.9 (2-C), 153.0 (3′-C, 5′-C), 152.7 (5-C), 140.7 (4′-C), 140.0 (3-C), 135.7 (C), 129.8 (CH), 128.6 (2CH), 127.2 (2CH), 126.1 (1′-C), 109.5 (10-C), 106.0 (6′-C, 2′-C), 95.9 (6-C), 92.5 (8-C), 72.0 (OCH_2_), 61.1 (4′-OCH_3_), 58.0 (CH_2_), 56.5 (7-OCH_3_), 56.4 (3′, 5′-2OCH_3_), 55.9 (5-OCH_3_), 53.3 (CH_2_), 52.8 (CH_2_), 47.2 (CH_2_), 41.7 (CH_2_), 28.1 (CH_2_), 22.9 (CH_2_). Anal. calcd for C_35_H_40_N_2_O_9_: C, 66.44; H, 6.37; N, 4.43%. Found: C, 66.22; H, 6.69; N, 4.11%.

**5k:** Yield, 82%, m.p. 123 ∼ 125 °C; IR: *ν*_max_ 1627, 1602, 1558, 1506, 1417, 1348, 1246, 1211, 1128, 1109, 1016, 852, 819; ^1^H NMR δ: 9.06 (1H, t, *J* = 4.0 Hz, PhH), 8.56 (2H, d, *J* = 2.3 Hz, PhH), 7.31 (2H, overlap *s*, 2’-H, 6’-H), 6.47 (1H, d, *J* = 2.3 Hz, 8-H), 6.33 (1H, d, *J* = 1.7 Hz, H-6), 4.02 (2H, t, *J* = 12.6 Hz, OCH_2_), 3.86–3.92 (15H, *m*, 5OCH_3_), 3.77 (2H, *m*, piperazine), 3.36 (2H, br, piperazine), 2.50 (2H, br, piperazine), 2.36 (2H, *t*, *J* = 14.9 Hz, CH_2_), 2.32 (2H, br, piperazine), 1.69–1.74 (2H, *m*, CH_2_), 1.58–1.62 (2H, *m*, CH_2_); ^13^C NMR δ: 174.1 (4-C), 165.2 (C=O), 164.1 (7-C), 161.1 (9-C), 158.9 (2-C), 153.0 (3′-C, 5′-C), 152.6 (5-C), 148.6 (2C), 140.7 (4′-C), 139.9 (3-C), 139.3 (2CH),127.6 (CH), 126.2 (1′-C), 119.7 (C), 109.5 (10-C), 106.0 (2′-C, 6′-C), 95.9 (6-C), 92.5 (8-C), 72.2 (OCH_2_), 61.0 (4′-OCH_3_), 57.9 (CH_2_), 56.5 (7-OCH_3_), 56.4 (3′, 5′-2OCH_3_), 55.9 (5-OCH_3_), 53.1 (CH_2_), 52.6 (CH_2_), 47.9 (CH_2_), 42.7 (CH_2_), 28.2 (CH_2_), 23.2 (CH_2_). Anal. calcd for C_35_H_38_N_4_O_13_: C, 58.17; H, 5.30; N, 7.75%. Found: C, 57.84; H, 4.91; N, 7.98%.

**5l:** Yield, 48.5%, m.p. 94 ∼ 96 °C; IR: *ν*_max_ 1627, 1600, 1558, 1506, 1411, 1344, 1246, 1211, 1163, 1130, 1016, 854, 815; ^1^H NMR δ: 8.14 (1H, d, *J* = 8.6 Hz, PhH), 7.50 (1H, dd, *J* = 2.3 and 2.3 Hz, PhH), 7.34 (1H, d, *J* = 2.3 Hz, PhH), 7.33 (2H, overlap *s*, 2′-H, 6′-H), 6.47 (1H, d, *J* = 2.3 Hz, 8-H), 6.33 (1H, d, *J* = 2.3 Hz, 6-H), 4.01 (2H, *t*, *J* = 13.1 Hz, OCH_2_), 3.89–3.94 (15H, *m*, 5OCH_3_), 3.73 (2H, *m*, piperazine), 3.17 (2H, *m*, piperazine), 2.54 (2H, br, piperazine), 2.35 (2H, *t*, *J* = 14.4 Hz, CH_2_), 2.27 (2H, br, piperazine), 1.68–1.72 (2H, *m*, CH_2_), 1.56–1.61 (2H, *m*, CH_2_); ^13^C NMR δ: 174.1 (4-C), 164.9 (C=O), 164.1 (7-C), 161.1 (9-C), 158.9 (2-C), 153.0 (3′-C, 5′-C), 152.6 (5-C), 143.6 (C), 141.3 (C), 140.7 (4′-C), 139.9 (3-C), 134.5 (CH), 129.9 (CH),128.3 (C), 126.4 (CH), 126.2 (1′-C), 109.5 (10-C), 106.0 (2′-C, 6′-C), 95.9 (6-C), 92.5 (8-C), 72.2 (OCH_2_), 61.0 (4′-OCH_3_), 58.0 (CH_2_), 56.5 (7-OCH_3_), 56.4 (3′, 5′-2OCH_3_), 55.9 (5-OCH_3_), 52.6 (CH_2_), 52.3 (CH_2_), 46.9 (CH_2_), 41.9 (CH_2_), 28.2 (CH_2_), 23.2 (CH_2_). Anal. calcd for C_35_H_38_ClN_3_O_11_: C, 59.03; H, 5.38; N, 5.90%. Found: C, 60.21; H, 5.75; N, 6.17%.

### 2.3. Anticancer assay

The MTT was purchased from Sigma. Cell Cycle Analysis Kit and Apoptosis Analysis Kit were purchased from Bestbio (China). Mitochondrial membrane potential assay kit with JC-1 was purchased from Beyotime (China). All the materials were used as received without further purification unless noted specifically. The cell culture was maintained on DMEM medium (Hyclone, US) supplemented with 10% fetal bovine serum (Sijiqing, China), 100 U/mL penicillin and 100 µg/mL streptomycin in 25-cm^2^ culture flasks at 37 °C humidified atmosphere with 5% CO_2_. Drugs were dissolved in DMSO and filtered through 0.22-µm pore-size filter units before addition to cell line appropriate media. When tumor cells were grown to log phase, diluting to 5 × 10^4^ cells mL^−1^ with the complete medium, 100 µL of the obtained cell suspension was added to each well of 96-well culture plates. The subsequent incubation was performed at 37 °C, 5% CO_2_ atmosphere for 48 h before subjecting to anti-proliferation assessment. After 48-h exposure period, 20 µL of PBS containing 5 mg·mL^−1^ of MTT was added to each well. After 4 h, the medium was replaced by 150 µL DMSO to dissolve the purple formazan crystals produced. The absorbance at 492 nm of each well was measured on an ELISA plate reader. The data represented the mean of three experiments in triplicate and were expressed as means ± SD. The IC_50_ value was defined as the concentration at which 50% of the cells could survive. The following human tumor cell lines were used in the assay: human gastric cancer cells SGC-7901, human liver cancer cell SMMC-7721 and HepG2, human cervical cancer cell Hela and human glioma cell U87.

### 2.4. Octanole water partition coefficients

Octanole water partition coefficients was measured by the method of shake flask with slight modification. The aqueous phase was replaced by PBS (pH 7.4). First, the phase of octanol and the aqueous were saturated with each other. Then, the mixture containing title compounds was shaken at 37 °C. After 24 h, the mixture was centrifuged at 4000 rpm for 30 min, followed by the measured with ultraviolet spectrophotometer. At last, logP values were calculated.

### 2.5. Animals and cell line

Kunming mice (SPF, male or female, 20 ± 2 g) were purchased from the experimental animal center of China Pharmaceutical University. Animals were housed in a temperature (22 ± 2 °C) and relatively humidity (50%)-controlled room on a 12-h light/dark cycle, given free access to food and water, and acclimatised for at least one week prior to use. All the animal experiments were performed in accordance with the Regulations of the Experimental Animal Administration issued by the State Committee of Science and Technology of China. Efforts were made to minimise the number of animals used and their suffering. Animals were maintained in accordance with the Guides of Center for Developmental Biology, Anhui Medical University for the Care and Use of Laboratory Animals and all experiments used protocols approved by the institutions’ subcommittees on animal care.

Cell line used for evaluation of the *in vivo* antitumor activity in this study included EAC (Ehrlich ascites carcinoma cell line). Cell line was purchased by the Shanghai Institutes for Biological Sciences, Chinese Academy of Sciences, and the cells were cultured in RPMI-1640 medium, which was supplemented with 10% heat-inactivated fetal bovine serum, 100 U/mL penicillin and 100 U/mL streptomycin and cultured in an atmosphere of 5% CO_2_ at 37 °C. Cells were collected for the experiments in the logarithmic growth phase. To establish the tumor-bearing mouse model, the cell line was harvested and inoculated subcutaneously into the right armpit region of the mice. On the 7th day, the tumor ascrites were obtained and washed with sterile PBS. Under sterile condition, the tumor ascrites were diluted with sterile nomal saline to 1 × 10^10^/L cell suspension. Tumor ascites were maintained *in vivo* in mice by transplantation of 0.2 ml of ascites (2 × 10^6^ cells) from the infected mice to the non-infected mice.

### 2.6. *In vivo* tumor model

SD rats, male, 120 ∼ 160 g. 120 rats were divided randomly into two groups containing 12 normal rats and 108 DEN model rats. Rats were gavaged daily with DEN 8 mg/kg, once a day, 6 days a week. Normal saline rats were given the same volume, molding to 10 weeks. 60 model rats were randomly divided into five groups, 12 rats in each group, continued to give DEN; normal rats were given normal saline, molding to 16 week, stopped giving DEN. Compound **5i** treatment group from the 10th week, 50 mg·kg^−1^ was orally given, once a day, for 10 consecutive weeks. Similar parts of left lobe of liver were taken and fixed by 4% polyformaldehyde solution, embedded in paraffin, sliced and HE stained, for histopathological observation.

### 2.7. Apoptosis analysis

Apoptosis was detected by flow cytometric analysis of annexin V staining. Annexin V-FITC/PI assay was performed according to previously described^29^. First, adherent SGC-7901 cells were harvested and suspended in the annexin-binding buffer (1 × 10^6^ cells/mL). Then, cells were incubated with V-FITC buffer and incubated with annexin V-FITC for 15 min and then stained by PI for 5 min at 4 °C in the dark, immediately analyzed by flow cytometry. The data are presented as biparametric dot plots showing PI red fluorescence versus annexin V-FITC green fluorescence.

### 2.8. MMP assay

#### 2.8.1. FCM analysis

Exponentially growing SGC-7901 cells were cultured in six-well plates (5 × 10^5^/well) and then treated with compound **5i** for 48 h. After incubation, adherent cells were detached with 0.5% trypsin. Next, the detached and suspended cells were harvested in the DMEM medium. Then, cells were stained with JC-1 in a humidified incubator with 5% CO_2_, for 0.5 h in the dark at 37 °C. At last, SGC-7901 cells were washed in JC-1 dyeing buffer twice and analyzed by a flow cytometer.

#### 2.8.2. Fluorescence staining

SGC-7901 cells were seeded into 6-well plates, then treated with compound **5i** at various concentrations for 48 h. Then, the cells were washed by PBS and stained by JC-1 in a humidified incubator with 5% CO_2_, for 20 min at 37 °C. After that the cells were washed with JC-1 buffer twice and finally visualised using a fluorescence microscope.

### 2.9. Western blot analysis

An equivalent volume of 5 × SDS-sample buffer was added to cell lysates and boiled for 10 min. The supernatants were subjected to electrophoresis on SDS-PAGE gels and transferred to PVDF membrane by using a Bio-Rad apparatus. Membranes were blocked for 3 h at room temperature in TBST containing 5% non-fat dried milk and probed overnight at 4 °C with antibodies. CyclinD1 (abcam, ab134175, 1:10000), CDC25A (abcam, ab156574, 1:1000), CDC25C (abcam, ab32051, 1:1000), bcl-2 (abcam, ab32124,1:1000), bax (abcam, ab32503, 1:1000), PARP (abcam, ab191217, 1:1000), γH2AX (abcam, ab81299, 1:5000), dyskerin (abcam, ab156877, 1:1000), NHP2 (abcam, ab180498, 1:1000), NOP10 (abcam, ab133726, 1:1000) antibody was performed with rabbit primary antibody. Otherwise, β-actin (ZSGB-BIO, 1:1000) detection was performed with mouse primary antibody which was served as internal controls. Protein bands of β-actin was visualised using peroxidase-labeled goat anti-mouse and others were visualised using peroxidase-labeled goat anti-rabbit secondary antibody (1:5000 dilution, 1 h, room temperature), and the protein expression signals were detected by the BeyoECL Plus (Beyotime).

### 2.10. Immunofluorescence staining

SGC-7901 cells were grown on glass coverslips in a 6-well culture plate until approximately 30–40% confluent and then treated with compound **5i** for 48 h. The coverslips were fixed in 4% paraformaldehyde for 15 min, followed by 0.5% Triton X-100 for 30 min at room temperature. Fixed and permeabilised cells were blocked with 3% BSA for 30 min at room temperature. Then, the cells were respectively immunolabeled with the appropriate primary and secondary antibodies. FITC-conjugated anti-rabbit IgG (ZSBIO, 1:100) for dyskerin emit green fluorescence. DAPI was used for nuclear staining.

### 2.11. Telomerase activity assay

Inhibit of telomerase activity was tested using the TRAP-PCR-ELISA assay. In detail, the SGC-7901 cells were firstly maintained in DMEM medium (GIBCO, New York) supplemented with 10% fetal bovineserum (GIBCO, NewYork), streptomycin (0.1 mg/mL) and penicillin (100 IU/mL) at 37 °C in a humidified atmosphere containing 5% CO_2_. After trypsinisation, 5 × 10^4^ cultured cells in logarithmic growth were seeded into T25 flasks (Corning, New York) and cultured to allow to adherence. The cells were then incubated with Staurosporine (Santa Cruz, Santa Cruz) and the drugs with a series of concentration as 60, 20, 6.67, 2.22, 0.74, 0.25 and 0.082 l g/mL, respectively. After 24-h treatment, the cells were harvested by cell scraper orderly following by washed once with PBS. The cells were lysed in 150 µL RIPA cell lysis buffer (Santa Cruz, Santa Cruz) and incubated on ice for 30 min. The cellular supernatants were obtained via centrifugation at 12,000 g for 20 min at 4 °C and stored at −80 °C. The TRAP-PCR-ELISA assay was performed using a telomerase detection kit (Roche, Basel, Switzerland) according to the manufacturer’s protocol. In brief, 2 µL of cell extracts were mixed with 48 µL TRAP reaction mixtures. PCR was then initiated at 94 °C, 120 s for predenaturation and performed using 35 cycles each consisting of 94 °C for 30 s, 50 °C for 30 s, 72 °C for 90 s. Then 20 µL of PCR products were hybridised to a digoxigenin (DIG)-labeled telomeric repeat specific detection probe. And the PCR products were immobilised *via* the biotin-labeled primer to a streptavidin-coated microtiter plate subsequently. The immobilised DNA fragment were detected with a peroxidase-conjugated anti-DIG antibody and visualised following addition of the stop regent. The microtitre plate was assessed on TECAN Infinite M200 microplate reader (Mannedorf, Switzerland) at a wavelength of 490 nm, and the final value were presented as mean ± SD.

### 2.12. Statistical analysis

Student's *t*-test was used to determine statistical significance at *p* < .05. SPSS 17.0 and Graphpad Prism 5 were used for analysis. All data were expressed as means ± SD.

### 2.13. General procedure for docking

Discovery Studio 2017 was used (Accelrys Software Inc., San Diego, California, USA). Crystal structure of telomerase TERT (PDB: 3DU6) was used as template. The active site was defined and sphere of 5 Å was generated around the active site pocket, with the active site pocket of BSAI model using C-DOCKER. The structure of protein, substrate were subjected to energy minimisation using CHARMm forcefield as implemented in DS 2017.

## 3. Results and discussion

### 3.1. Anticancer activity in vitro

In the screening assay studies, all synthesised compounds **5a**∼**5l** were evaluated for their activities against human glioma cell (U87), human cervical cancer cell (Hela), human liver cancer cell (SMMC-7721), human gastric cancer cell (SGC-7901) and human liver cancer cell (HepG2) cell lines. Compound ADM was used as the reference. From the Table 1, compound **5i** exhibited high activity against Hela, SMMC-7721, SGC-7901, U87 and HepG2 cell lines with IC_50s_ of 1.02, 1.33, 1.35, 2.50 and 4.12 µM, respectively, compared to the positive ADM. The preliminary SARs showed that most compounds had good activity against SGC-7901 cells (compounds **5f**, **5i**, **5j**, **5k**, **5l**), but, almost all compounds possessed poor activity against HepG2 cells. We then investigated the structure–activity relationships (SARs) profiles of the substituent group to sum up the rules. The prelimilary analysis indicates substituent **R** showed large effect for the activity, heterocycle is better than that benzene ring (compound **5i**). Further, the substituent number of benzene ring showed great influence on anticancer activity, among them, two substituents with different electronic groups are more beneficial to the activity (compounds **5k** and **5l**). Compared with the previous compounds, the title compounds with piperazine moiety have higher activity against gastric cancer cells, this pointed out the direction for us to further optimise this kind of skeleton structure.

**Table 1.** Anticancer activity *in vitro* of compounds **5a**∼**5l** against Hela, SMMC-7721, SGC-7901, U87 and HepG2 cell lines.^a^

Compound	IC_50_ (μM)^b^				
	Hela	SMMC-7721	SGC-7901	U87	HepG2
**5a**	25.51 ± 1.02	28.01 ± 0.98	16.61 ± 1.32	16.60 ± 1.95	33.40 ± 1.56
**5b**	11.33 ± 0.85	17.02 ± 0.99	10.45 ± 1.08	—^d^	23.40 ± 1.23
**5c**	9.77 ± 0.62	15.90 ± 1.01	9.31 ± 1.05	25.44 ± 1.55	28.00 ± 1.84
**5d**	31.41 ± 1.73	32.05 ± 1.53	18.51 ± 0.89	—^d^	35.51 ± 1.65
**5e**	25.11 ± 1.50	31.27 ± 1.63	13.25 ± 0.88	—^d^	—^d^
**5f**	14.40 ± 0.80	24.21 ± 1.44	6.97 ± 0.85	18.00 ± 0.66	37.44 ± 1.69
**5g**	17.03 ± 1.51	23.20 ± 1.83	10.49 ± 1.01	21.56 ± 0.90	40.45 ± 1.95
**5h**	22.37 ± 0.87	14.11 ± 1.27	9.22 ± 1.00	25.61 ± 1.40	35.30 ± 1.97
**5i**	1.02 ± 0.21	1.33 ± 0.14	1.35 ± 0.17	2.50 ± 0.11	4.12 ± 0.51
**5j**	8.30 ± 0.30	7.29 ± 0.82	7.56 ± 1.01	20.34 ± 1.25	—^d^
**5k**	2.94 ± 0.25	4.01 ± 0.38	5.12 ± 0.50	8.14 ± 0.22	21.20 ± 1.53
**5l**	3.80 ± 0.31	5.20 ± 0.87	5.81 ± 1.01	16.20 ± 1.45	29.12 ± 1.75
**ADM^c^**	0.21 ± 0.03	0.81 ± 0.06	0.98 ± 0.27	2.24 ± 0.12	2.87 ± 0.21

a Three experiments in triplicate.

b Inactive at 60 µM.

c ADM (doxorubicin) used as a control.

d None.

To evaluate the membrane permeability of active compounds, logP (o/w) which is an important parameter, was used to predict this profile. The logP values of compounds **5f, 5i, 5i** and **5 l** were 3.35, 2.81, 3.01 and 4.82, respectively (Table 2), which suggested that the active compounds are lipophilicity (logP < 5). Among them, compound **5i** show better hydrophilicity with membrane permeability.

**Table 2.** Log *p* values of some compounds.

Compound	Log *p*^a^
**5f**	3.35
**5i**	2.81
**5j**	3.01
**5l**	4.82

a Octanole water partition coefficients of some compounds were measured by the shake flask method with slight modification.

### 3.2. Assay of human normal cell

In order to determine the selective cell toxicity of selected compounds. We subsequently conducted a proliferative inhibition assay with human normal gastric mucosa cell (GES-1). As shown in Table 3, all compounds manifested obvious un-toxic effect on GES-1 with IC_50_ from 2.55 to 10.01 mM.

**Table 3.** Selected compounds against GES-1 proliferation^a^

Compound	GES-1 (IC_50_, mM)
**5a**	4.18 ± 0.60
**5f**	2.98 ± 0.55
**5e**	10.01 ± 1.22
**5i**	3.71 ± 0.33
**5k**	2.55 ± 0.38

a MTT assays were used for evaluation, and values were expressed as mean IC_50_ of the triplicate experiment.

### 3.3. Cell apoptosis

**Figure 2.**
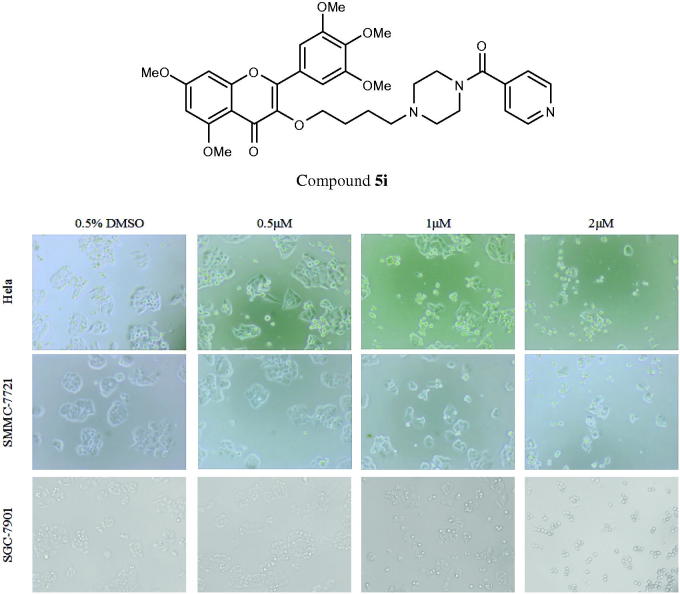
Cell morphology effects*^a^*. *^a^*Hela, SMMC-7721 and SGC-7901 cells were treated with compound **5i** at 0.5 μM, 1 μM and 2 μM for 48 h.

**Figure 3.**
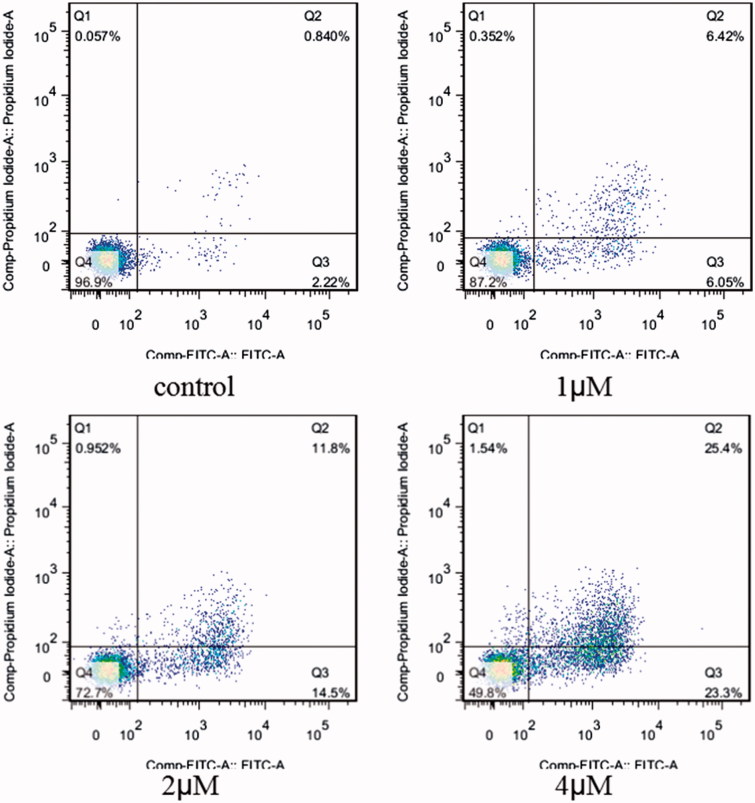
Apoptosis ratio detection. SGC-7901 cells were used for Annexin V/PI assay, treated with compound **5i** of 1 μM, 2 μM and 4 μM for 48 h, respectively; Analyses were performed at least three times, and a representative experiment was presented.

In order to determine whether above cell death induced by compound **5i** was due to apoptosis, cell apoptosis was investigated in this study. Because phosphatidylserine exposure usually precedes loss of plasma membrane integrity in apoptosis, the presence of AnnexinV+/PI-cells can be considered as an indicator of apoptosis. With the increase concentration of the compound **5i**, cell viability significantly decreased (SMMC-7721, SGC-7901 and Hela). Meanwhile, apoptosis and fragmentation were found (Figure 2). The results were given in Figure 3. Apoptosis ratios increased gradually with concentration manner. The apoptosis ratios were found to 12.47% (1 µM), 26.3% (2 µM) and 48.7% (4 µM), respectively, while that of control was 3.06%. So, compound **5i** could induce SGC-7901 cells apoptosis.

### 3.4. Prelimilary mechanisms of apoptosis

**Figure 4.**
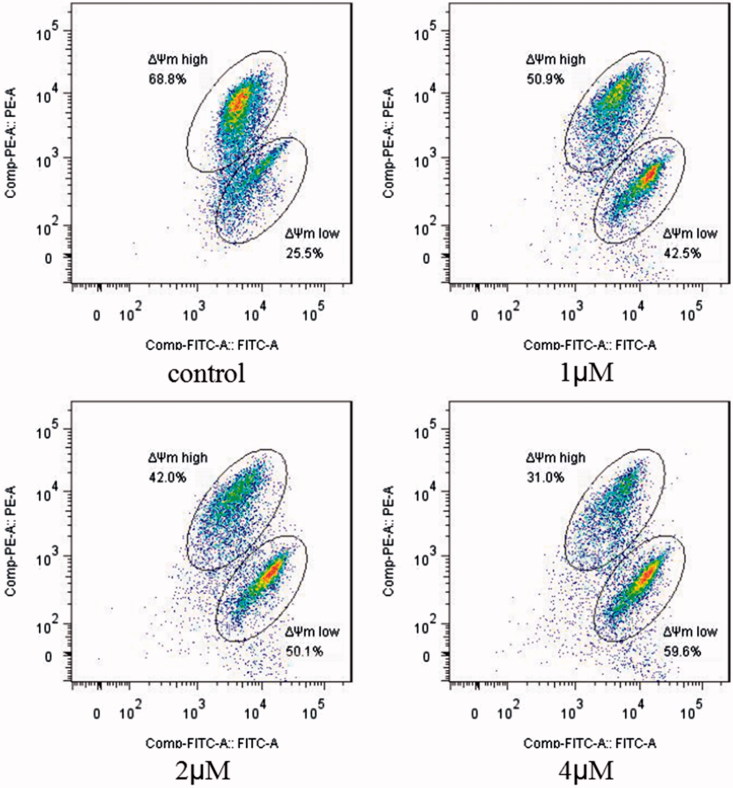
MMP assay. SGC-7901 cells were treated with compound **5i** of 1 μM, 2 μM and 4 μM for 48 h, respectively; Analyses were performed at least three times, and a representative experiment was presented.

**Figure 5.**
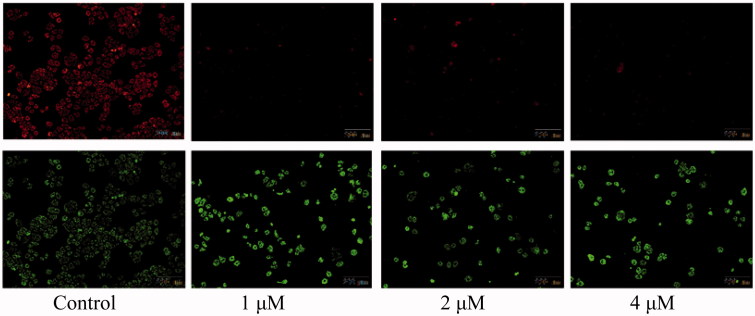
Collapse of mitochondrial membrane potential. SGC-7901 cells were treated with compound **5i** of 1 μM, 2 μM and 4 μM for 48 h, respectively, and stained by JC-1. Selected fields indicated the corresponding cells, among them, apoptotic cells (green), (orange-red).

**Figure 6.**
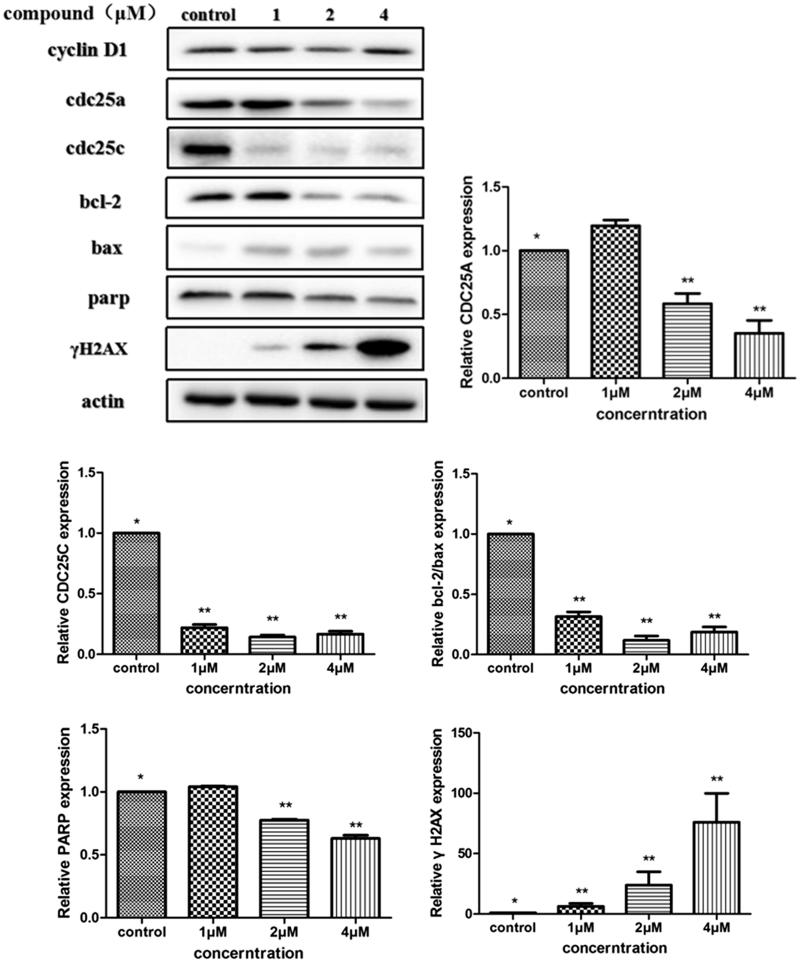
Determine the translation of proteins by western blot. SGC-7901 cells were treated with compound **5i** of 1 μM, 2 μM and 4 μM for 48 h, respectively. Significant difference between each concentration of compound **5i** and the control were shown as *p* < 0.05 (*) and *p* < 0.01 (**).

The above analysis showed that compound **5i** could induce cancer cells apoptosis. Losing of mitochondrial membrane potential MMP (ΔΨ) has been considered as a vital process for apoptosis. To investigate the role of mitochondria in the apoptosis treated with compound **5i**, the effect of this compound on MMP was measured by flow cytometry. In this test, SGC-7901 cells were stained with JC-1 (Figure 4). Compared with the control, treatment with compound **5i** for 48 h, the number of cells emitting red fluorescence (high ΔΨ) significantly decreased while the number of treated cells emitting green fluorescence (low ΔΨ) obviously increased (Figure 5), this resuts suggested the disruption of MMP might be participate in apoptosis induced by compound **5i**. As we know, the Bcl-2 combined with function of dyskerin is a key protein family in mitochondrial apoptosis pathway. So we next assessed the proteins related apoptosis (Bax/Bcl-2 and PARP). After treatment with compound **5i**, the proportion of Bcl-2/Bax and PARP were reduced (Figure 6). To investigate the mechanisms involved in DNA damage repair induced by compound **5i**, the expression of cell cycle proteins (cyclin D1, CDC25A and CDC25C) and DNA damage repair proteins γH2AX were also assessed by western blot. The results showed that compound **5i** could significantly decrease the expressions of CDC25A, CAC25C and γH2AX, which also reduced the expression of Bax/Bcl-2 and PARP. These effects may be achieved by inhibiting dyskerin and thus affecting the translation of mRNAs. This results supported our prelimilary anticipation that compound **5i** could regulate the cell cycle in phase of G2/M and induced the apoptosis through mitochondria pathway.

### 3.5. Expression changes of dyskerin-NHP2-NOP10

**Figure 7.**
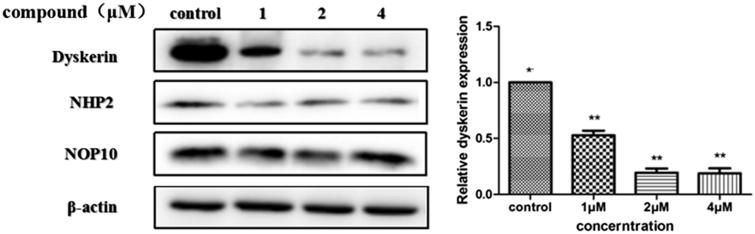
Determine the translation of dyskerin-NHP2-NOP10 by western blot. SGC-7901 cells were treated with compound **5i** of 1 μM, 2 μM and 4 μM for 48 h, respectively. Significant difference between each concentration of compound **5i** and the control were shown as *p* < 0.05 (*) and *p* < 0.01 (**).

In order to further investigate whether compound **5i** induced apoptosis through regulating dyskerin. Treatment with compound **5i** at different concentrations for 48 h (SGC-7901 cells were selected), protein level of dyskerin significantly reduced (Figure 7). At the same time, NHP2^30^ and NOP10^31^, as the components of the dyskerin-NHP2-NOP10 trimer, have also been assessed. But, no significant changes were observed. Dyskerin over-expression linked to a variety of tumor types has been reported^32^. There is no doubt that dyskerin expression plays an essential role in cancer-specific telomerase activation^33–34^. These results indicated compound **5i** may be an efficiency regulator as dyskerin protein.

### 3.6. Detection dyskerin

**Figure 8.**
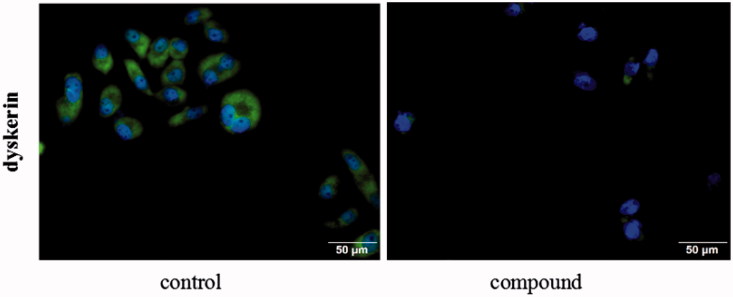
Detection the localisation of dyskerin through immunofluorescence. SGC-7901 cells were treated with compound **5i** of 2 μM for 48 h.

Staining with SGC-7901 cells, focused on dyskerin protein, the cellular localisation of dyskerin was studied by immunofluorescence. From the Figure 8, dyskerin was observed both in the cytoplasm and nucleus at the untreated SGC-7901 cells. After treatment with compound **5i** of 2 µM for 48 h, almost all the nucleus dyskerin was disappeared, and the cytoplasm localisation dyskerin also significantly reduced. This result revealed that compound **5i** could block dyskerin transport into the nucleus, thus lead to affecting assembly of the telomerase complex.

### 3.7. Telomerase inhibit activity

Dyskerin-NOP10-NHP2 trimer protein is the core component of telomerase, which plays a key role in the stability, activation and assembly of telomere. Basd on above, the compound **5i** can inhibit the expression of dyskerin, we want to see whether it has inhibitory activity against telomerase in the next study. So, selected compounds were assayed for their inhibition activities against telomerase (SGC-7901 cells extracts were used), BIBR1532 as a reference (Table 4). Among them, compounds **5i** showed strong telomerase inhibitory activity with IC_50_ value of 0.89 µM, comparable to that positive control BIBR1532. There was a good correlation between the activity *in vitro* and the IC_50_ of telomerase inhibitory.

**Table 4.** Inhibitory acticity of some compounds against telomerase

Compound	IC_50_ (µM) telomerase^a^
**5a**	11.33 ± 0.99
**5e**	13.29 ± 1.25
**5i**	0.89 ± 0.17
**5k**	1.44 ± 0.28
**BIBR1532^b^**	0.31 ± 0.10

a Telomerase supercoiling activity.

^b^BIBR1532 as a control.

### 3.8. Anticancer activity *in vivo*

**Figure 9.**
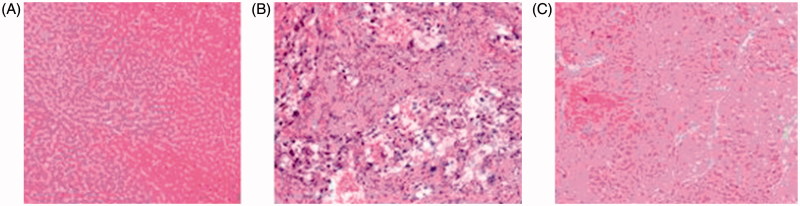
Anticancer activity *in vivo*. Compound **5i** against pathological changes (HE staining ×200). (A). Control (B). DEN Model (C). Compound **5i***^a^* *^a^*Animals were randomly divided into 3 groups (*n* = 12 per group).

In order to reveal the pathological effect of compound **5i***in vivo*, treatment with compound **5i**, the histopathology hepatic tumor was explored (DEN-induced rat was used). Compared to the model group, as we expected in the control group, hepatic lobular architecture and hepatic nuclei were clearly observed (Figure 9(A)). In the DEN model group, normal liver lobular structure has been completely destroyed, tumor cells showed evident atypia and low differentiation, larger nuclei and nucleoli were also observed (Figure 9(B)). In experimental group, compound **5i** could markedly abated pathological changes of hepatic lobules. The hepatic cells showed evidently reduced atypia, full cytoplasm and high differentiation. So, compound **5i** could significantly improve pathological changes for the rat of hepatic tumor (Figure 9(C)).

## 4. Conclusions

In brief, 12 new chromen-4-one derivatives as potential dyskerin regulators were designed and synthesised. Results of anticancer activity showed that some compounds had better antitumor activity both *in vivo* and *in vitro*. Among them, compound **5i** could significantly improve pathological changes. A preliminary mechanism indicated that this compound could induce gastric cancer cells apoptosis by arresting cell cycle, breaking mitochondria function, resulting in inhibiting telomerase activity. Meanwhile, western blot and immunofluorescence detection revealed that compound **5i** could dramatically decrease expression of dyskerin. These results preliminary show that this compound can regulate the expression of dyskerin and finally inhibit the activity of telomerase.

## Supplementary Material

Supplemental Material
