# Supplementary figures and images for: Discovery of new chromen-4-one derivatives as telomerase inhibitors through regulating expression of dyskerin
Source: J Enzyme Inhib Med Chem. 2018 Aug 22;33(1):1199–211. doi: 10.1080/14756366.2018.1466881 (PMC6104605; doi:10.1080/14756366.2018.1466881)

## Supporting information

### NMR of compound 5a~5l

5a:

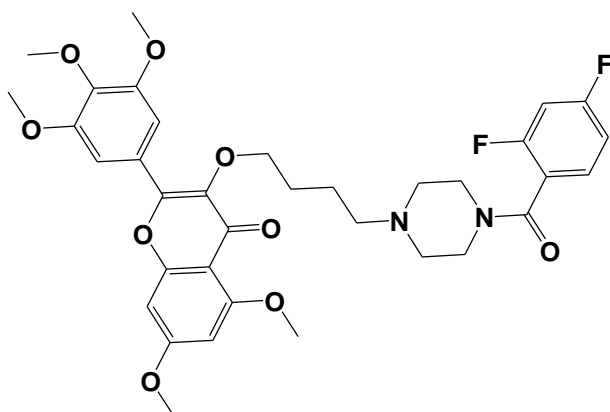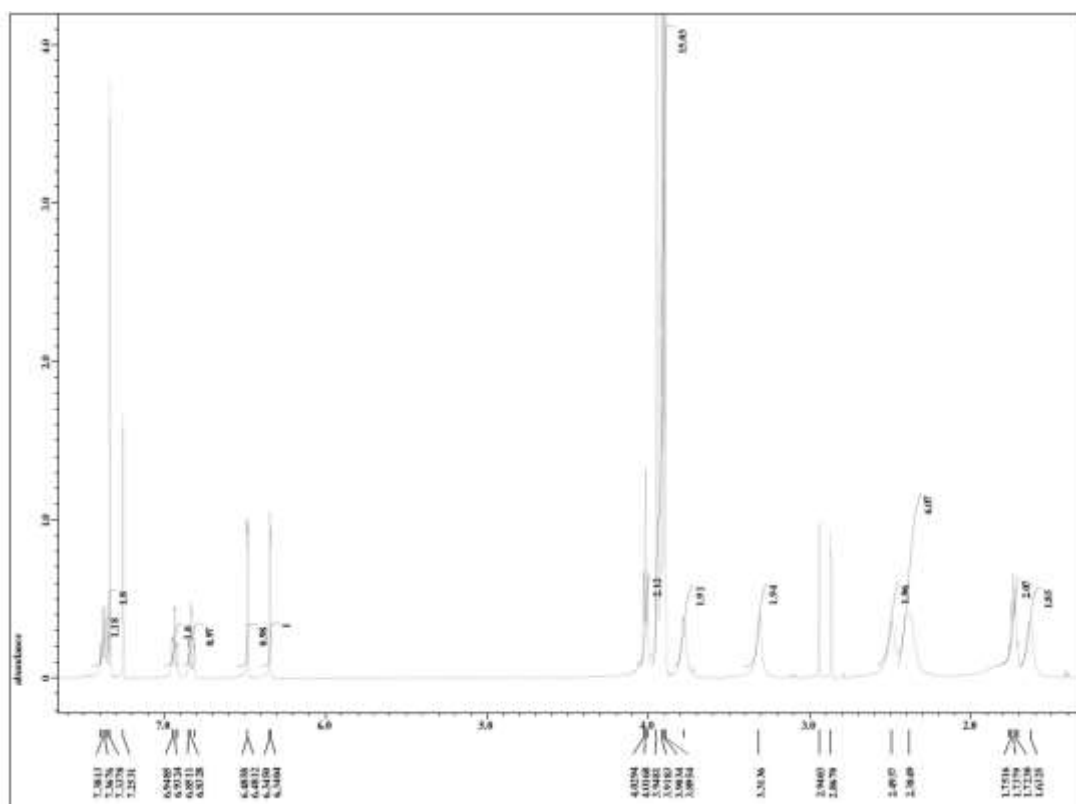

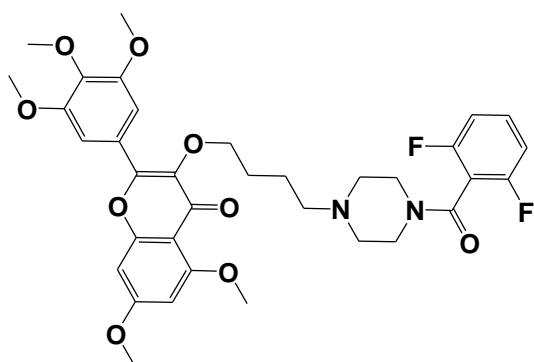

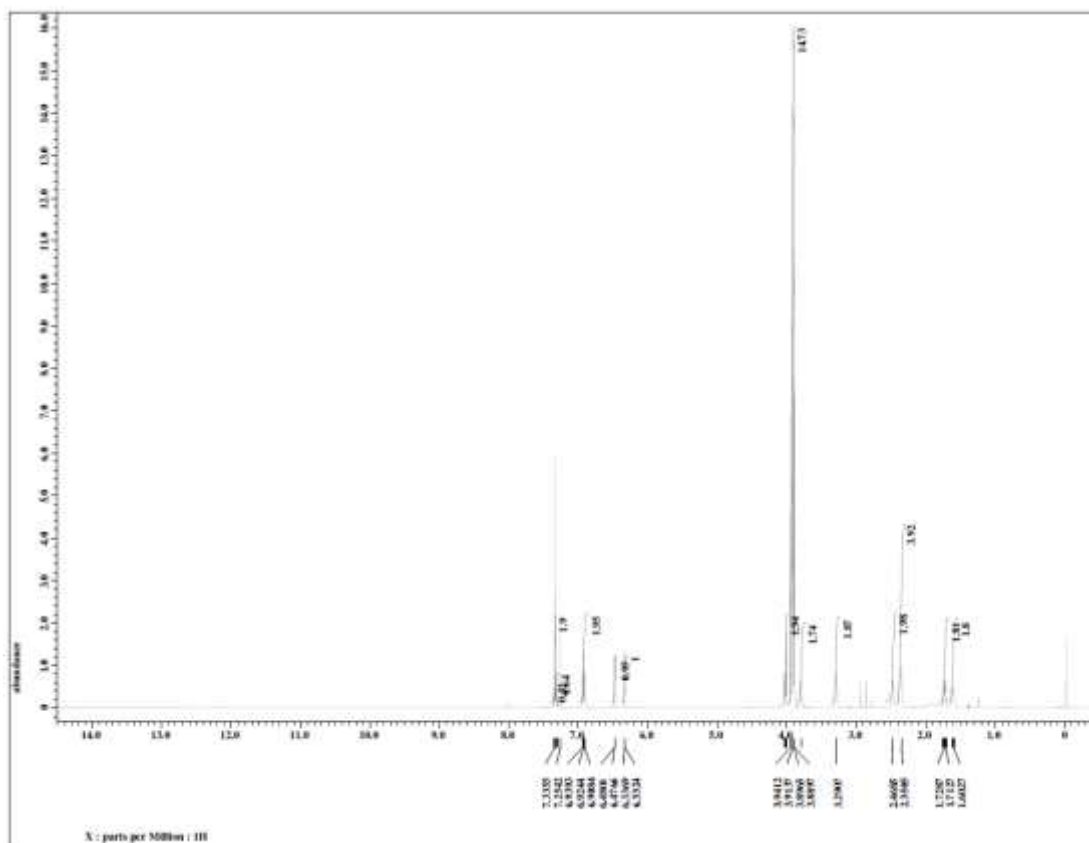

5c:

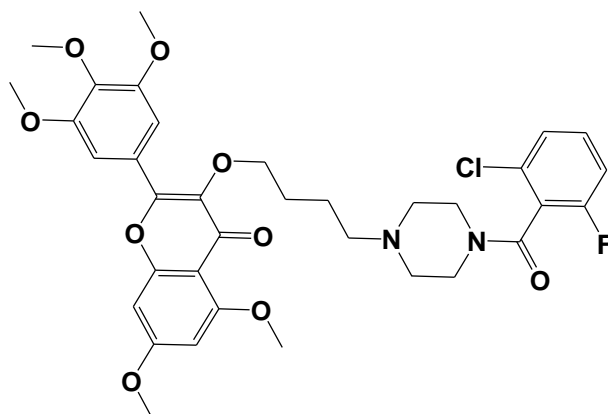

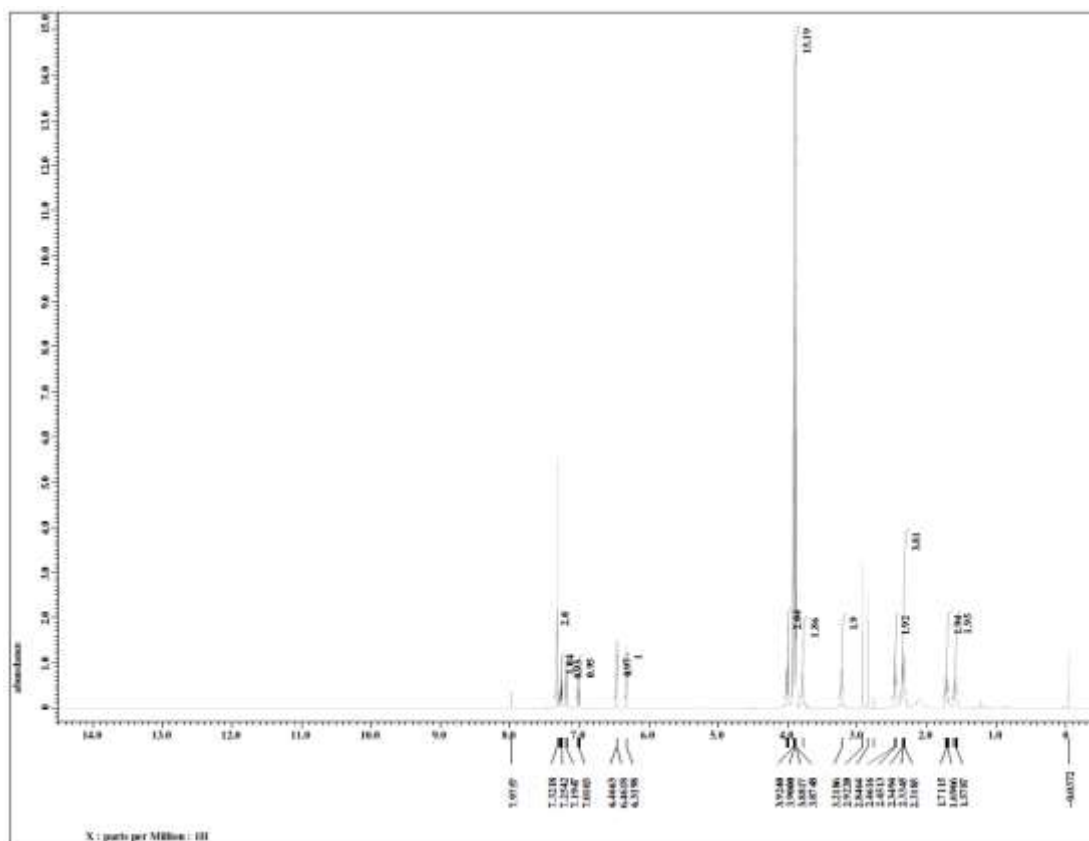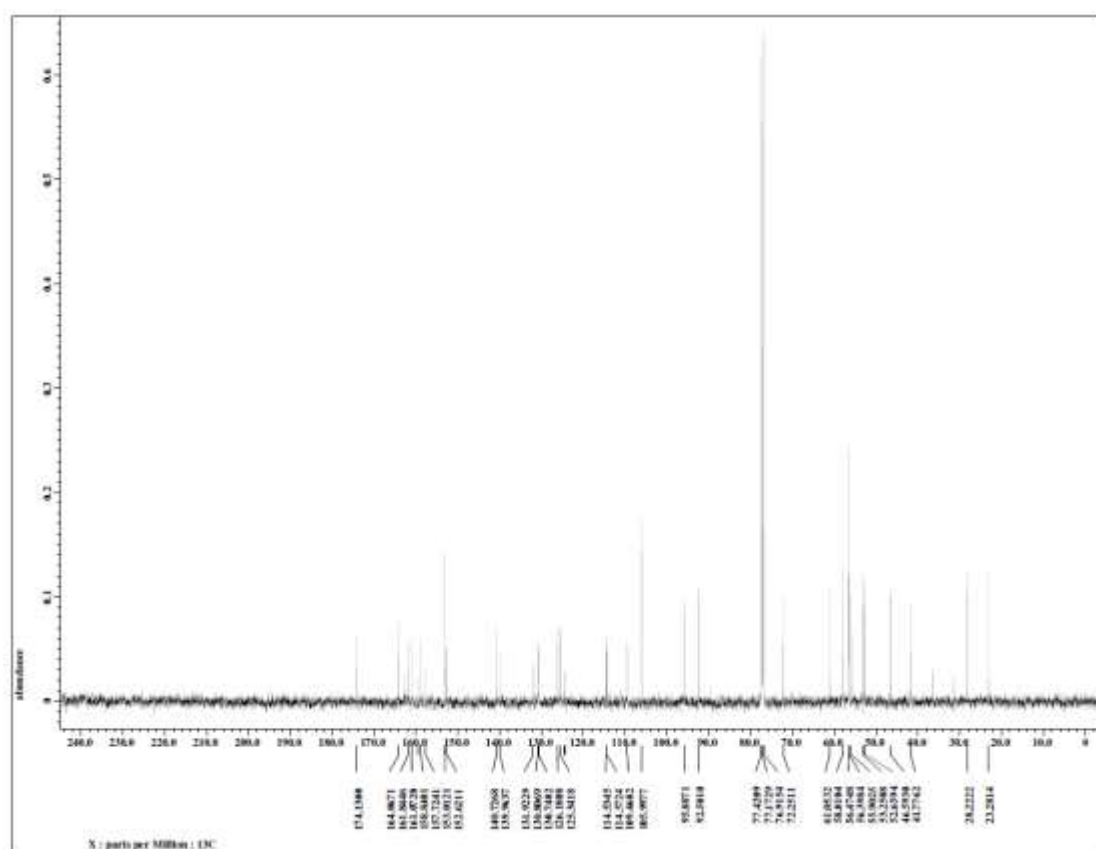

5d:

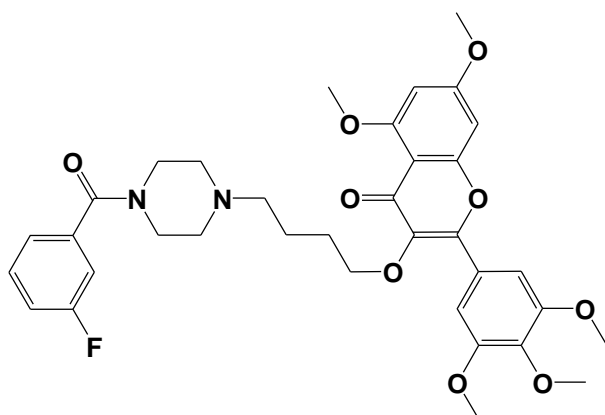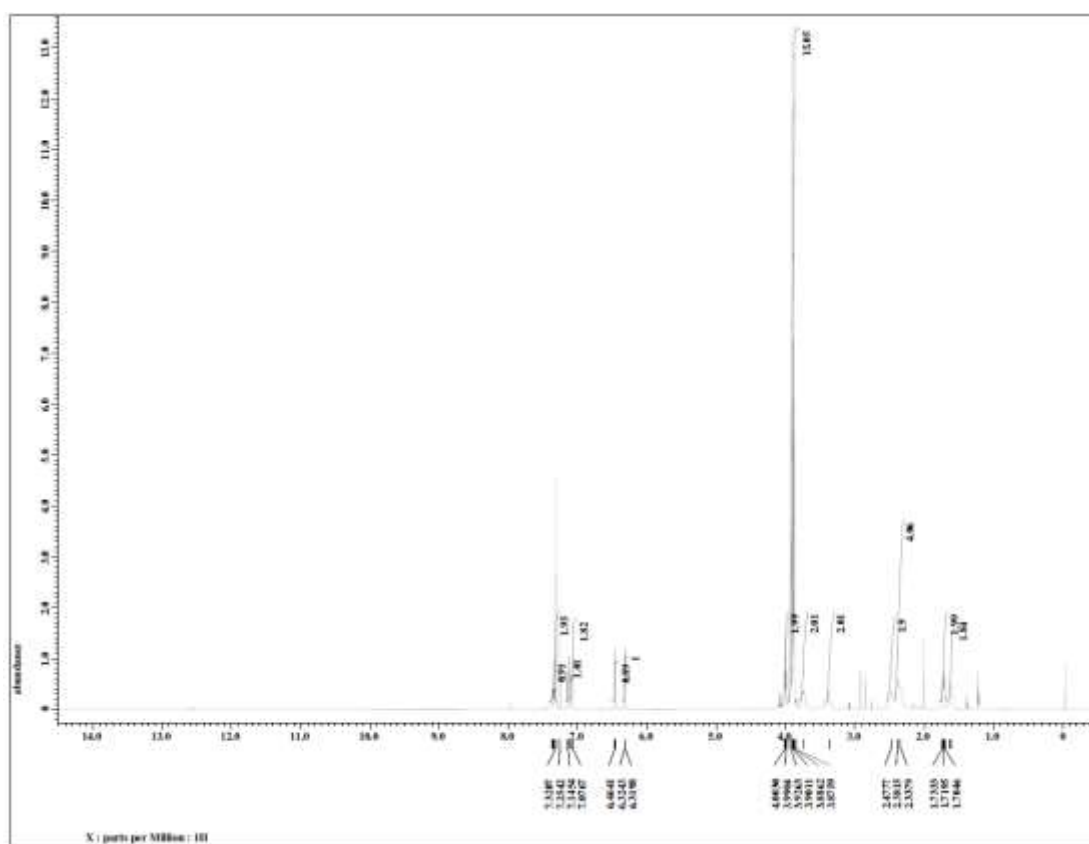



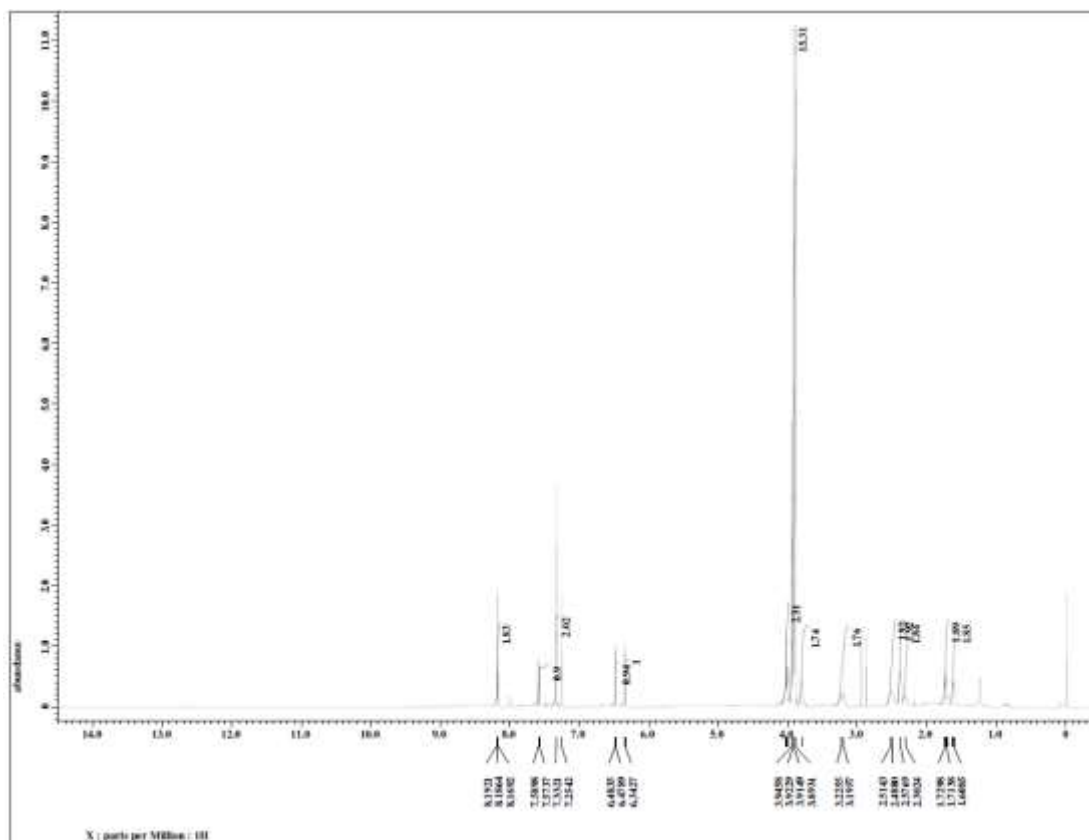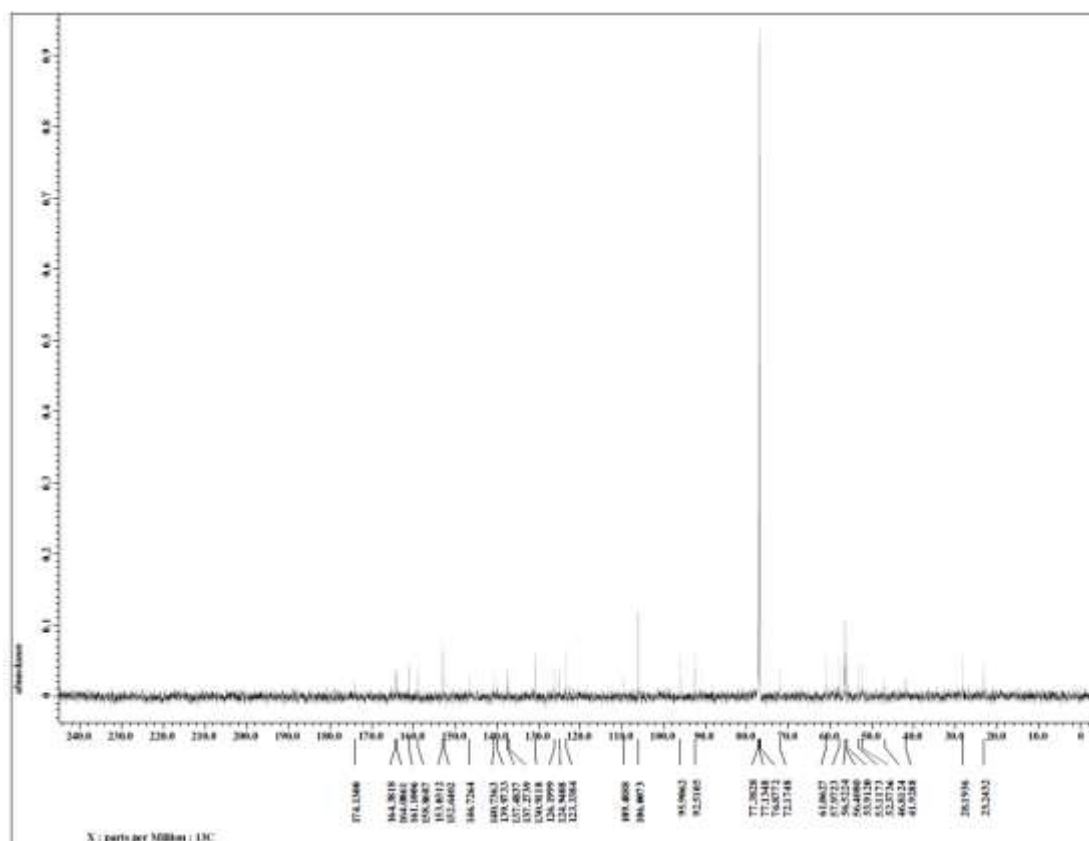

5f:

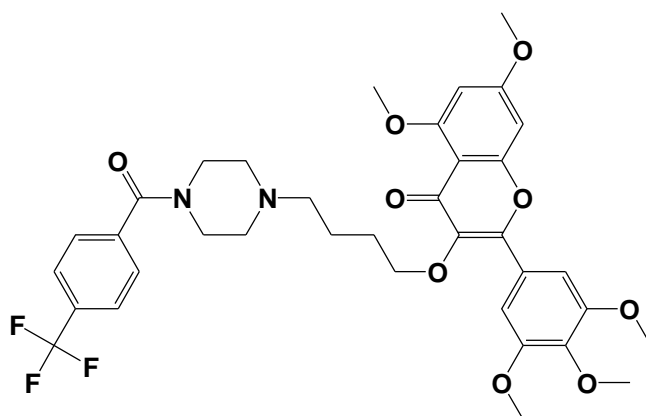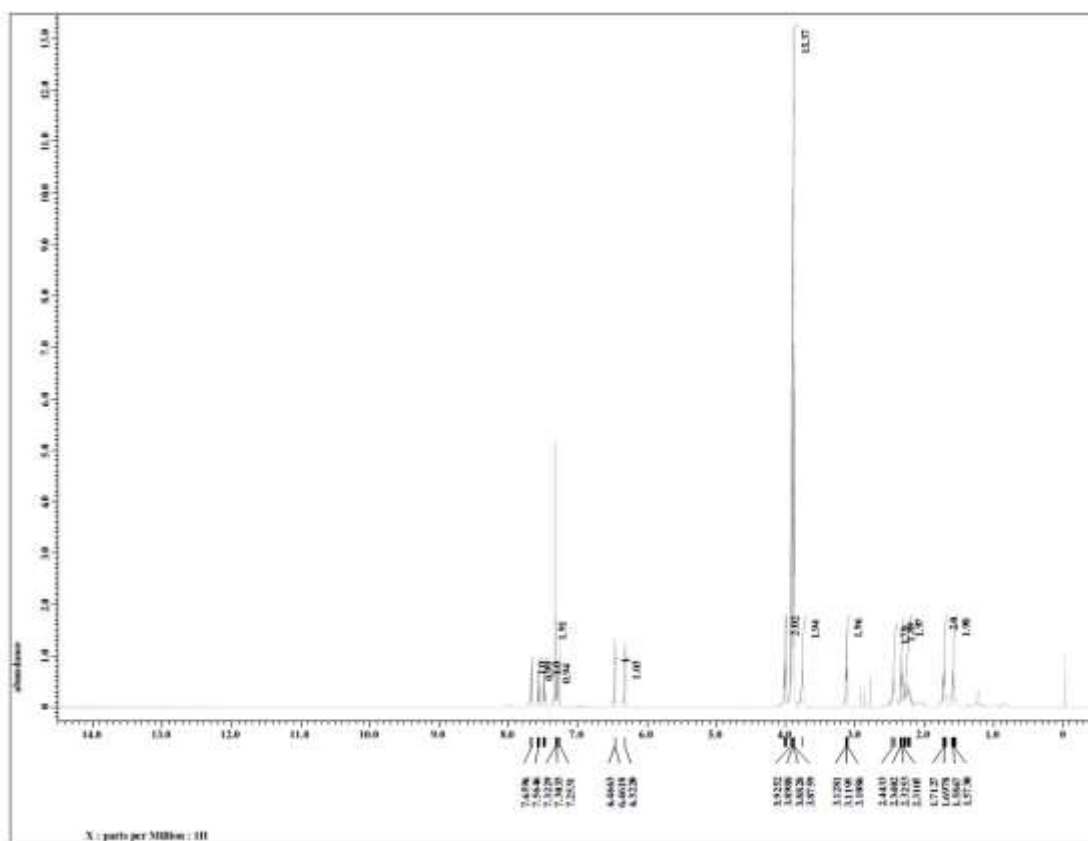

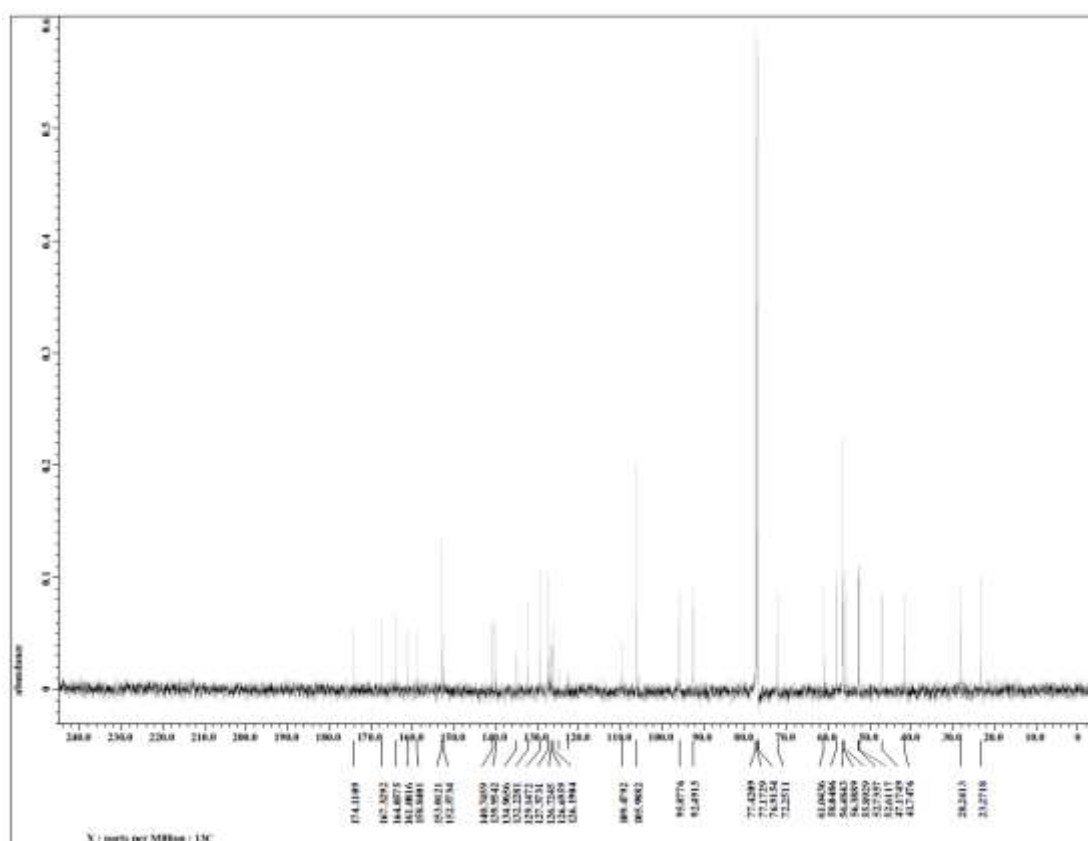

5g:

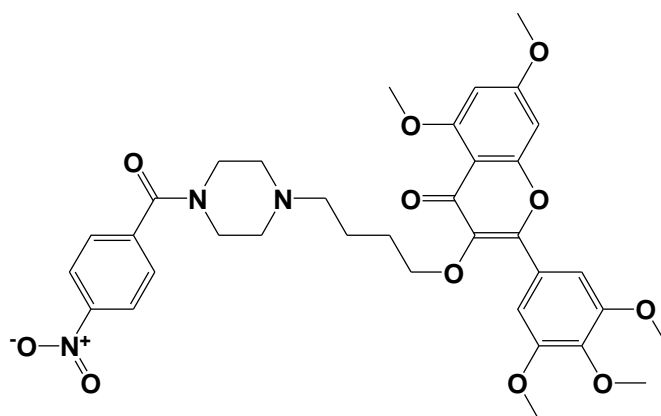

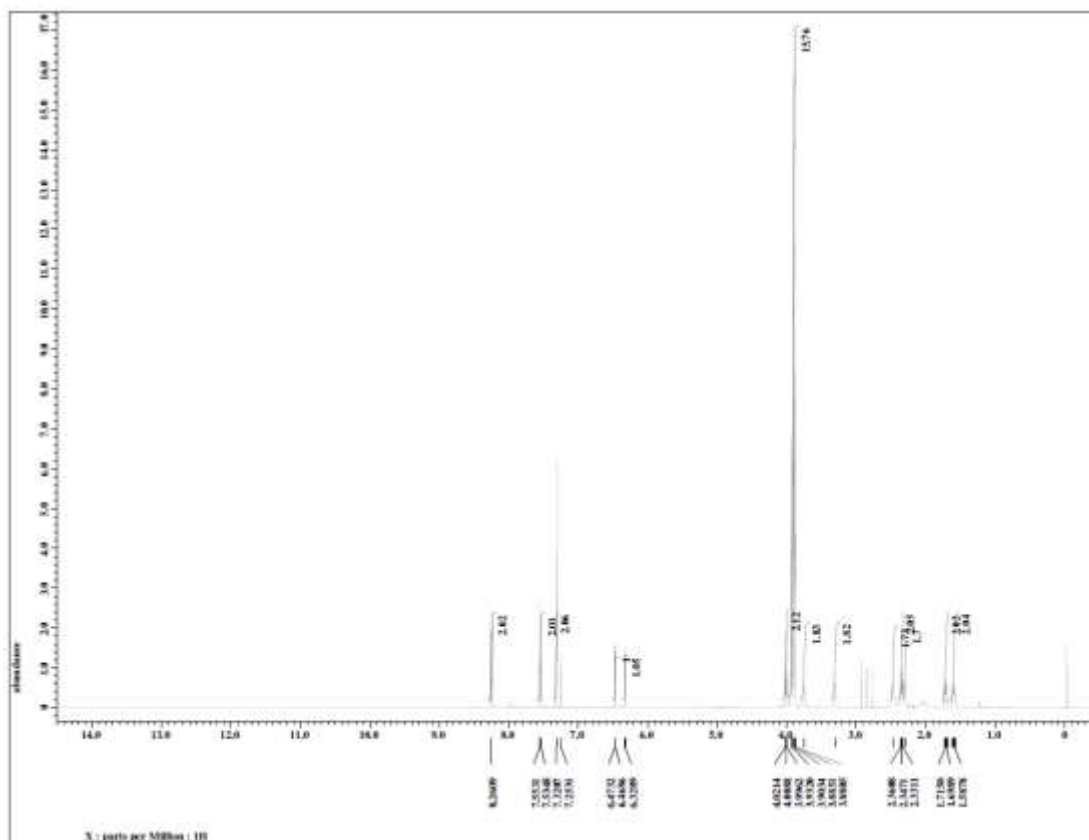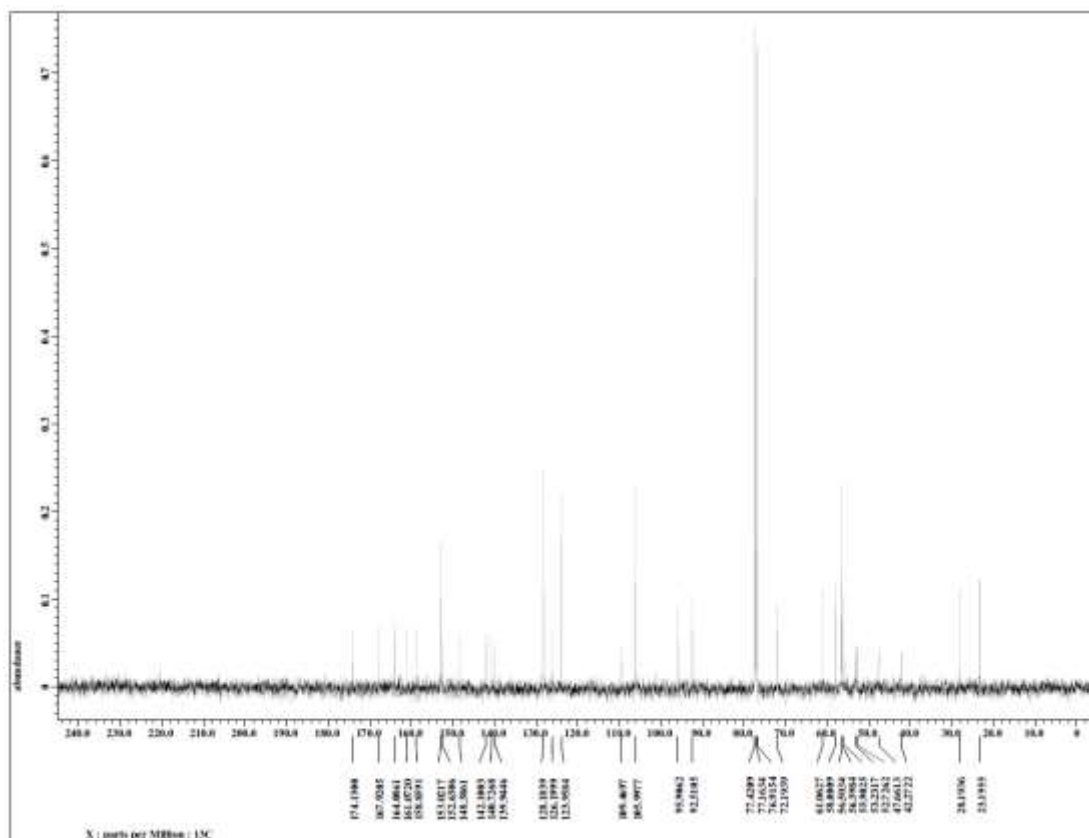

5h:

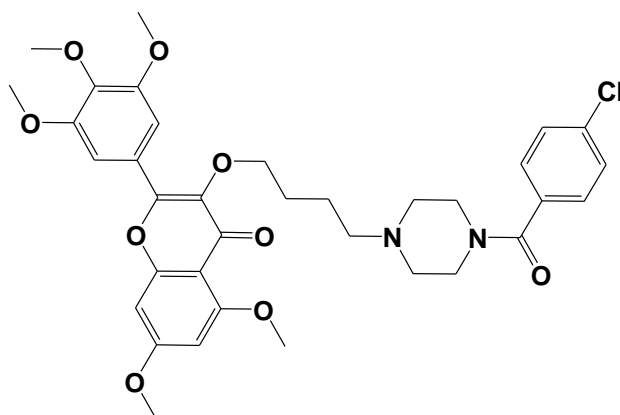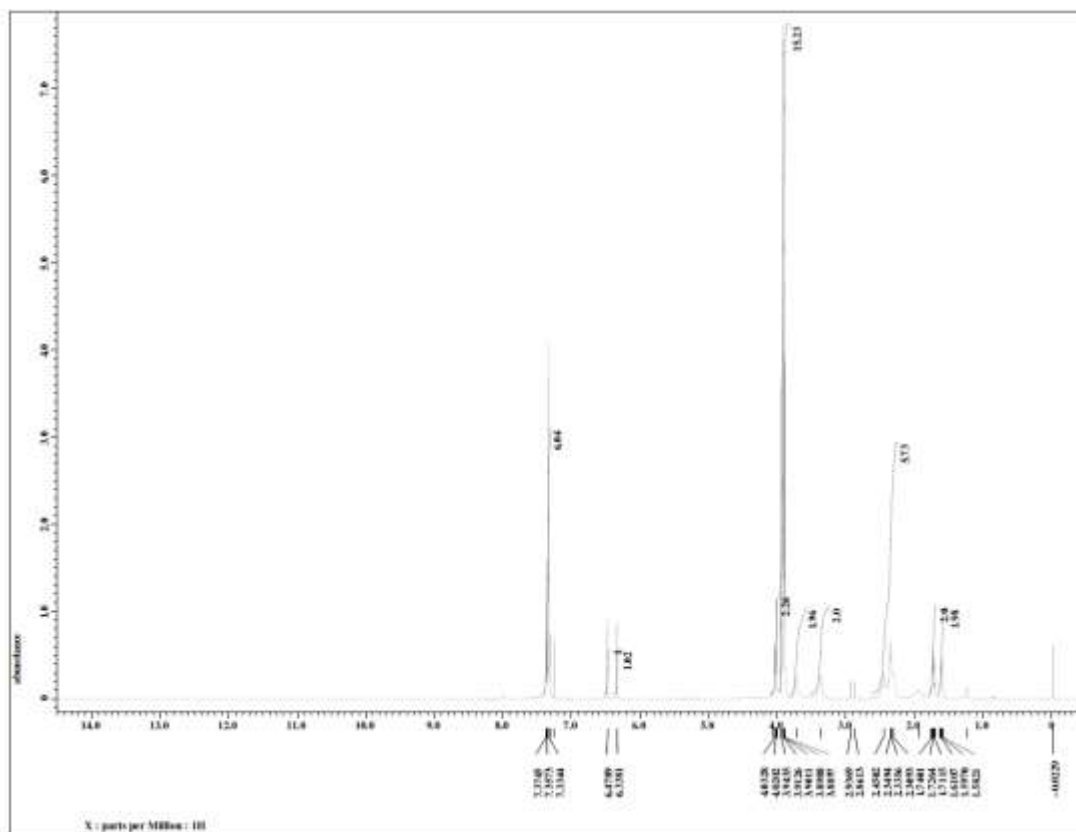

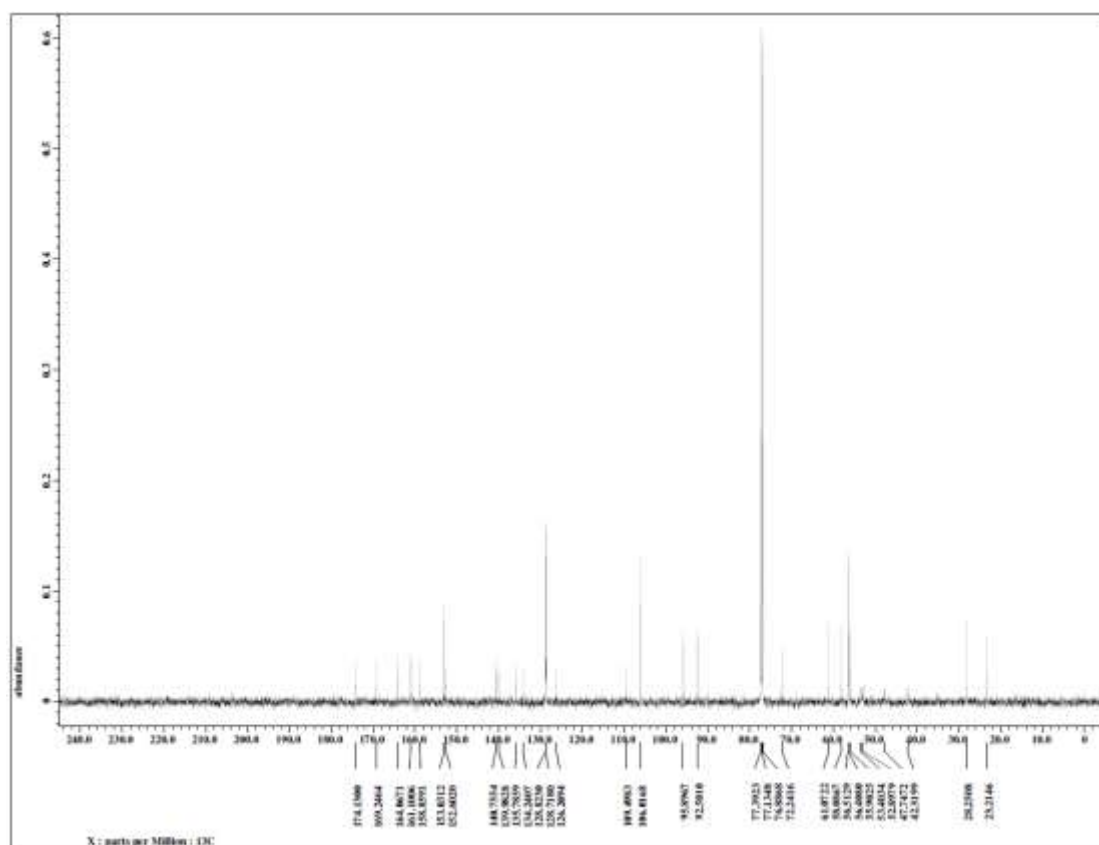

5i:

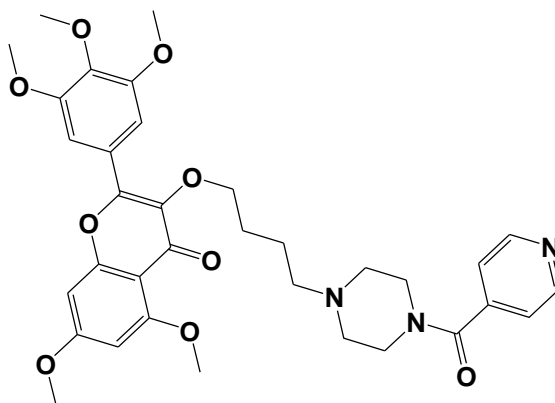

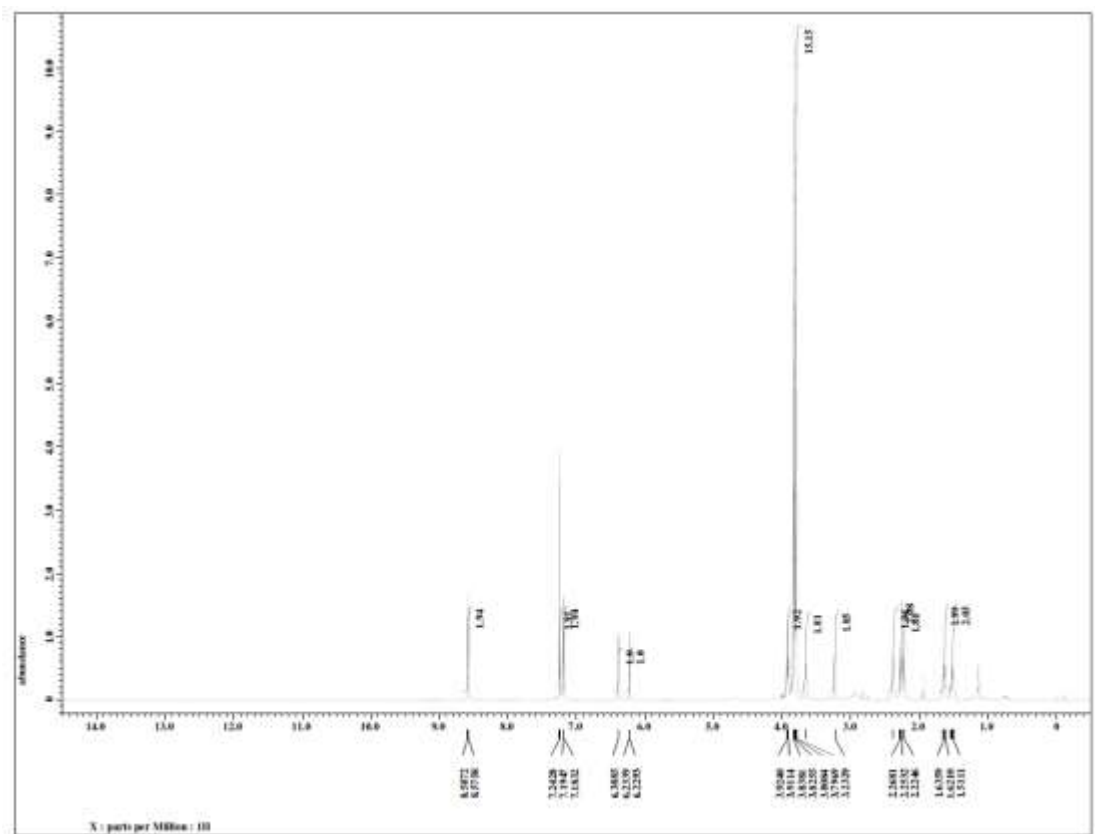

5j:

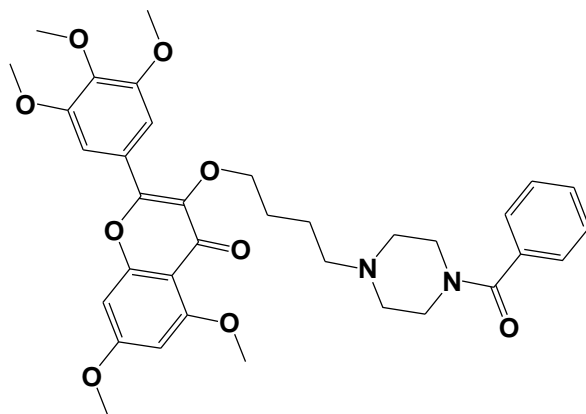

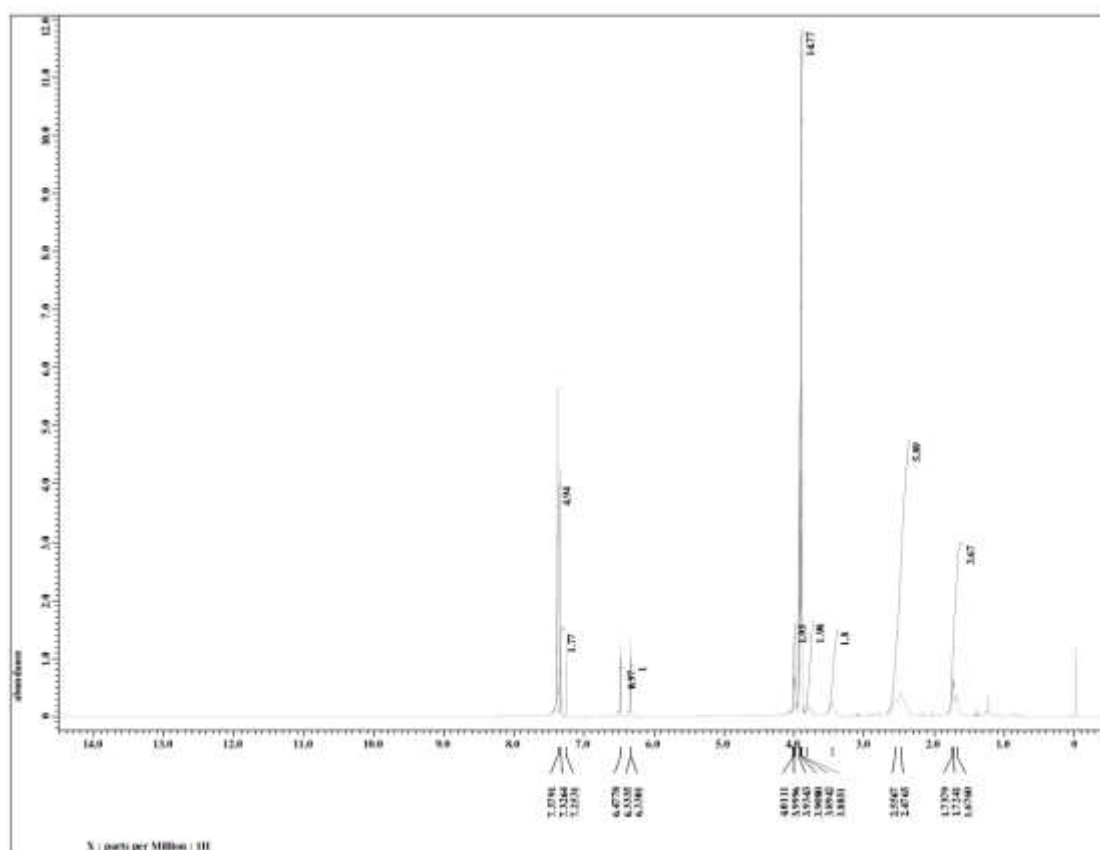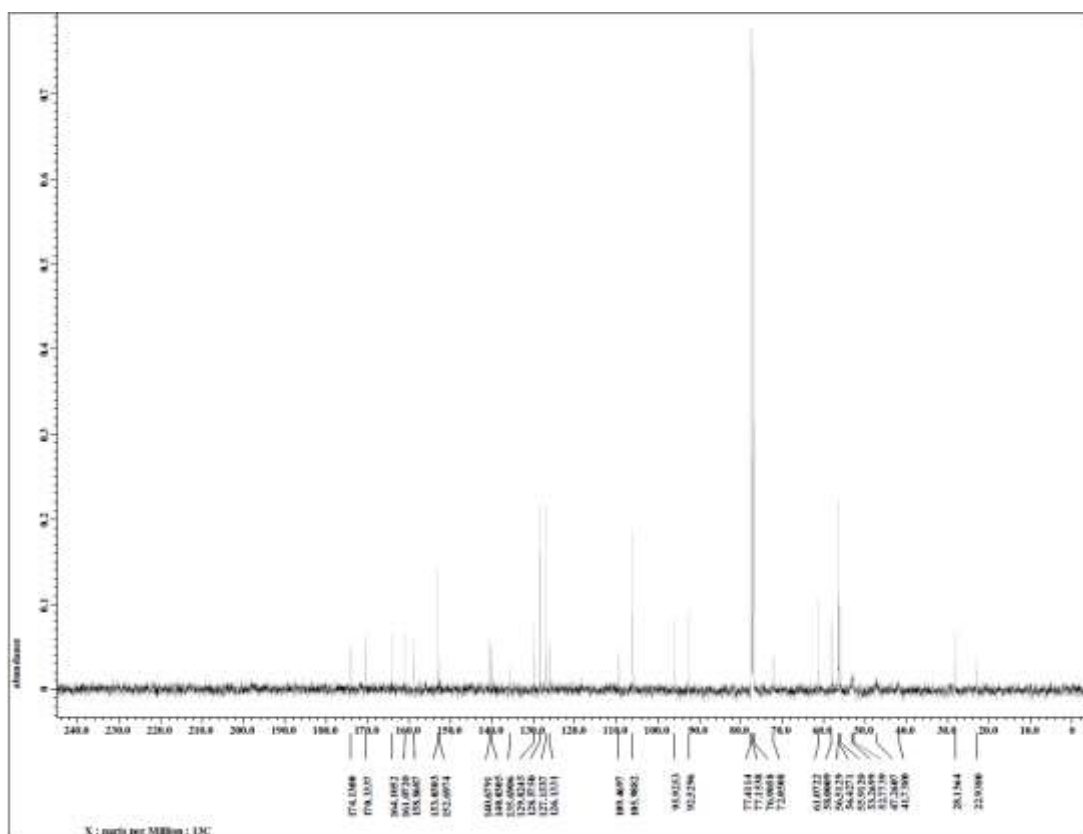

5k:

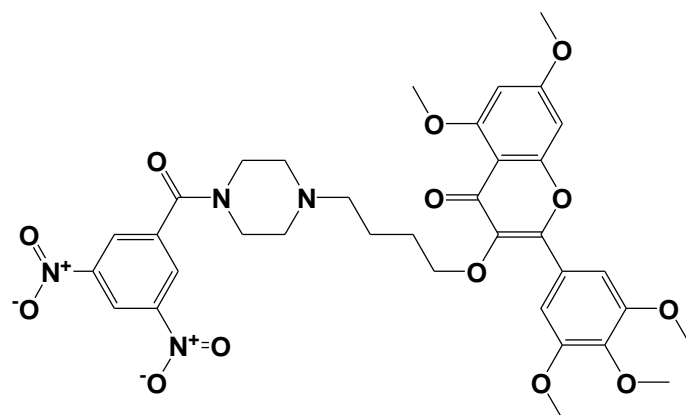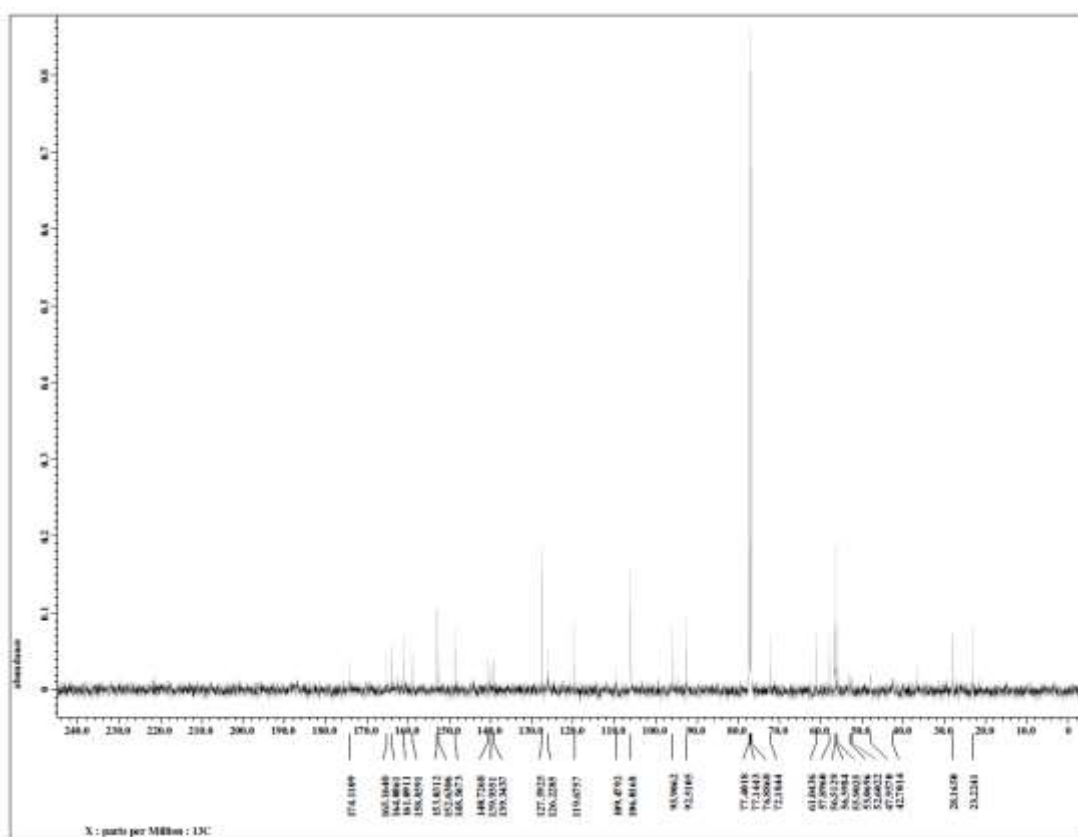

5l:

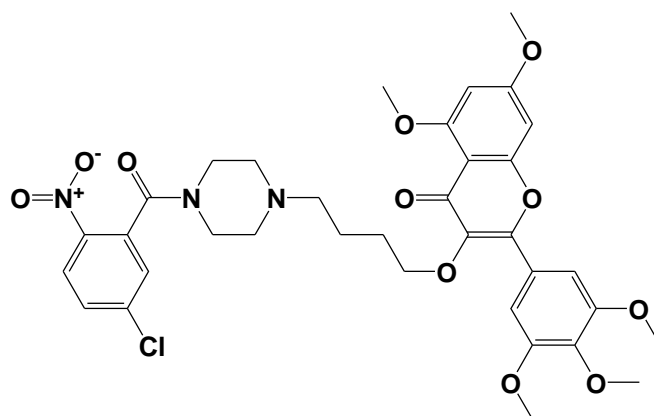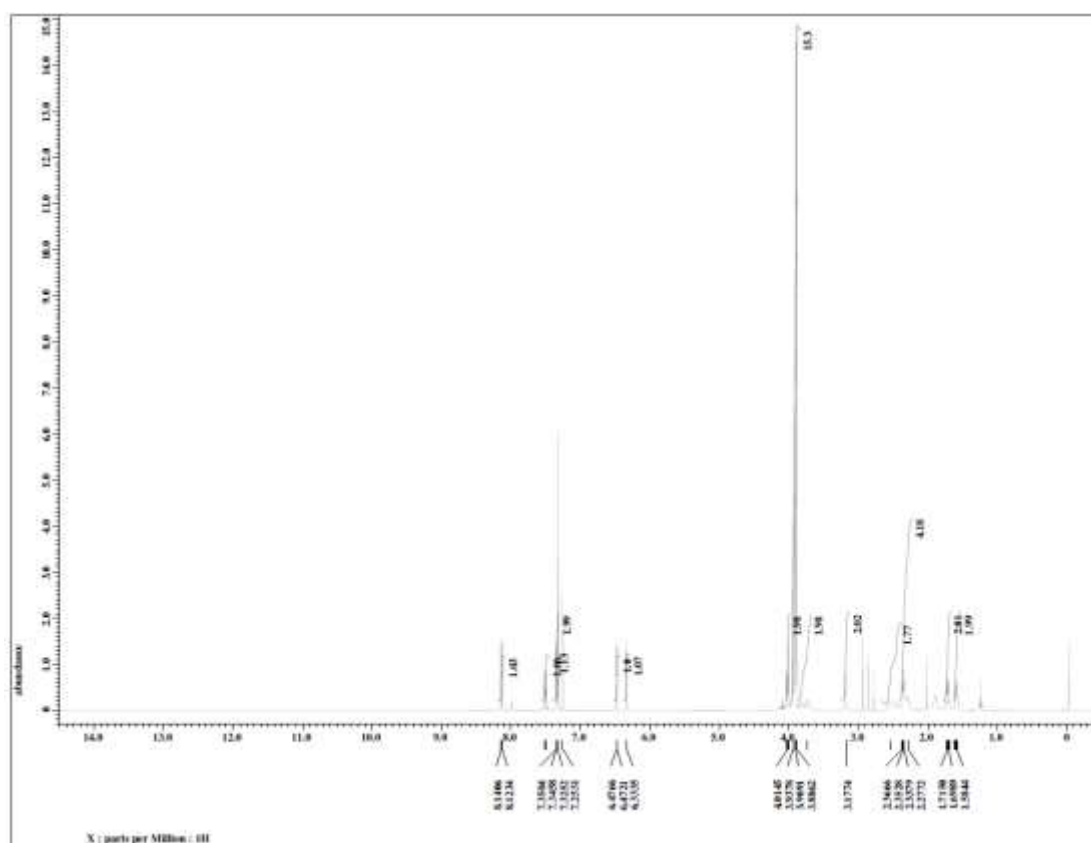

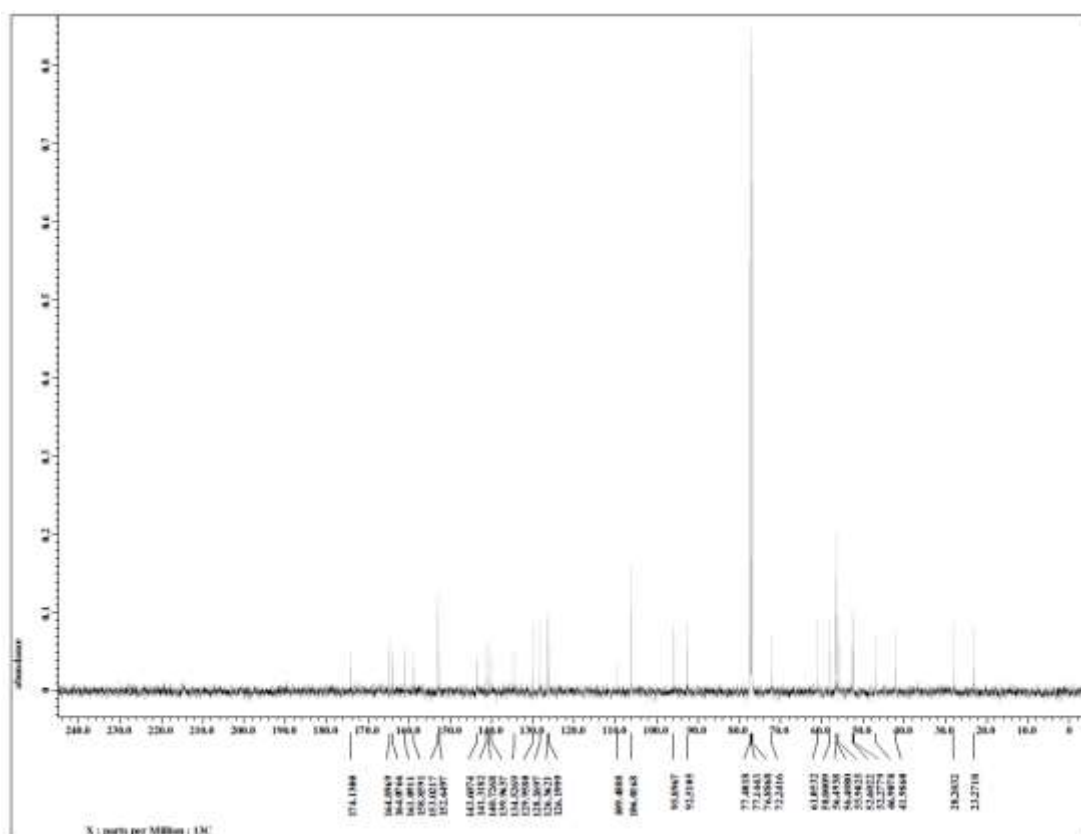

Supplement: Supplemental Material [file IENZ_A_1466881_SM4808.pdf]
